# Optimizing efficiency in the acute care chain: a systematic review on the implementation and impact of interdisciplinary short-term monitoring in acute care units

**DOI:** 10.1007/s11739-025-04194-w

**Published:** 2025-11-20

**Authors:** Tobias R. Neijzen, Mark E. Haaksma, Niels Raaijmakers, Suzanne Schol-Gelok, Hilde M. Wesselius, Marco Goeijenbier, Karin A. H. Kaasjager, Marjolein N. T. Kremers

**Affiliations:** 1 Department of Intensive Care, Spaarne Gasthuis, Haarlem, The Netherlands; 2 Department of Internal Medicine, Spaarne Gasthuis, Haarlem, The Netherlands; 3 Department of Internal Medicine and Emergency Department, St. Jans Gasthuis, Weert, The Netherlands; 4 Emergency Department, Albert Schweitzer Hospital, Dordrecht, The Netherlands; 5 Department of Internal Medicine, IJsselland Hospital, Capelle Aan Den IJssel, The Netherlands; 6 Department of Intensive Care, Erasmus University Medical Center, Rotterdam, The Netherlands; 7 Department of Infection and Immunity, University Medical Centre Utrecht, Utrecht, The Netherlands; 8 Julius Centre for Health Sciences and Primary Care, University Medical Centre Utrecht, Utrecht, The Netherlands; 9 Emergency Department, Erasmus University Medical Center, Rotterdam, The Netherlands; 10 Department of Health Services Research and CAPHRI School for Public Health and Primary Care, Ageing and Long Term Care, Maastricht, The Netherlands

**Keywords:** Acute medical units, Organisation of acute care, Acute care unit (ACU), Acute care for the elderly (ACE), Short stay unit (SSU), Acute medical unit (AMU)

## Abstract

The growing number of patients with multimorbidity and increasing disease complexity presenting to the Emergency Department (ED) poses a global challenge, with overcrowding as a major consequence. To address these pressures, various interdisciplinary short-term monitoring and treatment units, such as Acute Medical Units, Frailty Units, Observation Units, and Chest Pain Units, have been implemented to improve patient flow from presentation to discharge. This systematic review, conducted according to PRISMA guidelines, aimed to evaluate the reported effects of these units on patient outcomes and ED crowding. We searched multiple databases and included studies reporting at least one primary outcome measure (length of stay, mortality, costs, readmissions, or staff satisfaction) following implementation of such a unit. The search yielded 2972 results, of which 50 studies met the inclusion criteria. Reported outcomes included reductions in hospital length of stay (0.24 to 6.16 days), cost savings (ranging from €162 to nearly €2 million across eight studies), and stable or decreased mortality in some settings. However, the evidence base is heterogeneous, with units differing substantially in patient populations, aims, and models of care, and cost analyses often relying on limited methods. Consequently, strong general conclusions across all unit types are not possible. Nonetheless, these findings suggest that when well-matched to local system bottlenecks and patient populations, specialized acute care units can improve patient flow and contribute to the accessibility of emergency care without compromising quality.

*Trial Registration*: 10.17605/OSF.IO/A9EF7

**Supplementary Information:**

The online version contains supplementary material available at 10.1007/s11739-025-04194-w.

## Introduction

Growing patient numbers with multimorbidity and increased disease complexity presenting to the Emergency Department (ED) is a globally important challenge [1, 2]. Multimorbidity and high case complexity contribute significantly to ED overcrowding, which in turn is associated with prolonged waiting times, higher mortality, and increased risk of adverse outcomes [1–6]. Although health expenditure in high-income countries continues to rise, hospital resources such as beds and staff have not kept pace with growing demand. OECD data show a steady decline in hospital beds per capita across most member countries since 2011, while demand from ageing, multimorbidity, complex care needs, and rising emergency presentations continues to increase. This mismatch means the issue is less about reduced funding and more about demand outstripping system capacity, resulting in real-world shortages at the point of care [7].

For this reason, several strategies are being implemented to improve patient flow from initial presentation until discharge [8–16]. One of the strategies is the implementation of Acute Care Units (ACUs). These are interdisciplinary short-term monitoring and treatment units, aimed at improving patient-centered outcomes while reducing healthcare costs, shortening hospital stay and improving admission flow [17–20].

While the concept of ACUs has been proven to be an effective strategy, it has undergone significant evolution over the years. Various types of acute observation or admission units have emerged to specialize in meeting the unique needs of various patient populations or hospital settings. For instance, acute admission units for older patients (Acute Care for Elderly units; ACE) or acute units designed for brief observation (Short Stay Units; SSU) [21, 22]. The wide range of terms and definitions used in the literature can make it challenging to interpret an overview article. To support clarity, we have, therefore, included an explanatory table (Supplementary file 2) that allows readers to look up the different forms of ACUs while reading this manuscript.

However, definitions, implementation, and evaluation of ACUs remain inconsistent, and reported clinical and financial benefits vary across healthcare settings, patient populations, and unit models [11, 19, 20, 23–29]. Notably, a recent Cochrane review found no randomized controlled trials evaluating the impact of acute assessment services on ED patient flow compared with standard ED-only care, underscoring a significant evidence gap despite their widespread adoption [30]. The aim of this systematic review is therefore to summarize the available evidence to support more effective implementation of ACUs.

## Methods

In this systematic review, the PRISMA (Preferred Reporting Items for Systematic Reviews and Meta-Analyses) guidelines were followed. The protocol was prospectively registered in OSF registries under 10.17605/OSF.IO/A9EF7.

### Search strategy

**Fig. 1:**
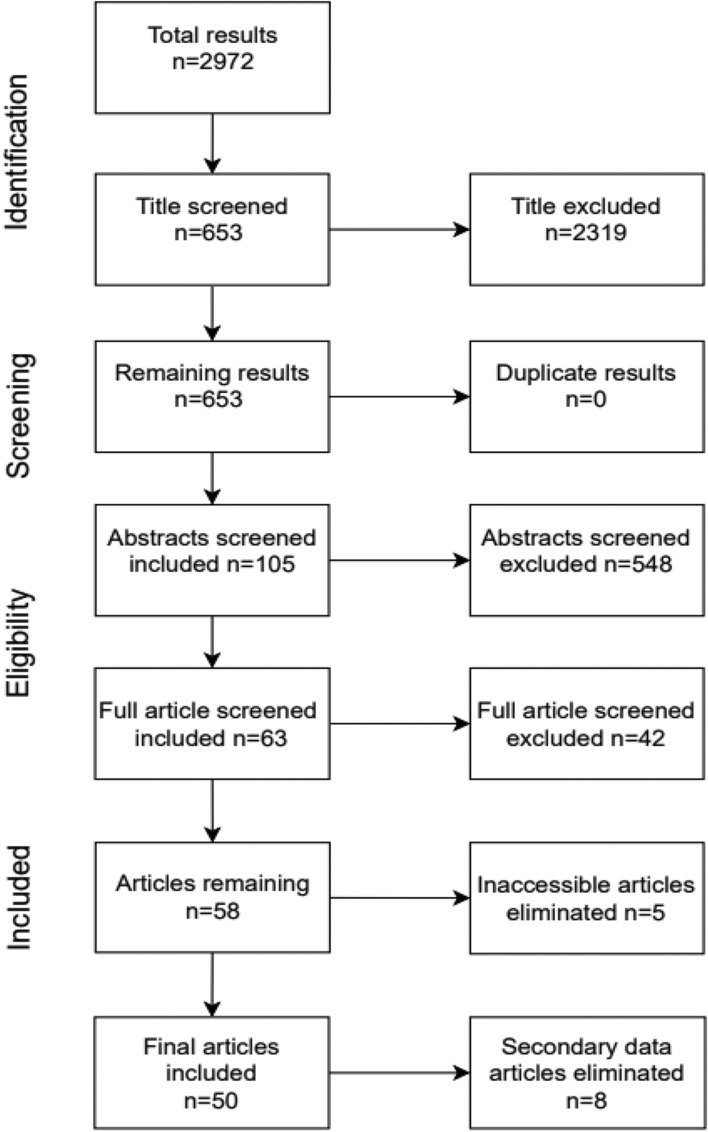
Flowchart of study selection process

The literature search for this systematic review was performed by a medical librarian (JE) on 1–5-2024. It was performed in Medline via PubMed, EMBASE and the Cochrane Database of Systematic Reviews by using search terms such as ‘acute medical unit’, ‘observation unit’ or ‘short stay unit’. The complete search strategy is described in search string Fig. 1 (Supplementary file 1).

### Selection of studies

Studies eligible for inclusion were those conducted in designated hospital wards specifically staffed and equipped to receive patients presenting with acute illness from emergency departments and/or the community. These wards provide expedited multidisciplinary and/or medical specialist assessment, care, and treatment for up to a designated period, typically between 24 and 72 h, before discharge or transfer to medical wards. An overview with commonly used terms and definitions is provided in Supplementary2 (S2). Intensive Care Units, Medium Care Units, Neuro/Brain Care Units and Cardiac Care Units were excluded from this review.

Eligible studies needed to report either patient-centered outcomes, such as hospital length of stay, mortality, and patient satisfaction, or organizational outcomes, including cost analysis, staffing numbers, and readmission rates. There were no restrictions on the type of study. Studies written in English were eligible for inclusion.

### Inclusion of studies

Search results were imported into Rayyan; a web and mobile app for systematic reviews to remove duplicates and facilitate effective screening [31]. Titles and abstracts were screened by a first reviewer (TN,NR,SG,HW) and a second reviewer (MK,KK). Consequently, full-text analyses were performed by two independent reviewers (TN,MH). In case of disagreement on study inclusion, resolution was achieved by consensus meetings with a third reviewer (MG). References of the included studies were further screened to identify other eligible studies by snowballing.

### Quality of studies

All information from the included studies is presented in Supplementary file 3, and study quality was assessed using the GRADE approach.

## Results

Details regarding the study selection process are summarized in Fig. 1. Of the initial 2972 identified publications, 50 studies were included. These comprised 3 randomized controlled trials (RCTs), 34 retrospective studies, 4 before-and-after studies, 2 case studies, 3 observational studies, 1 questionnaire-based study, 2 systematic reviews, and 1 Cochrane review.

The majority of studies were conducted in Europe (17), followed by the USA (12), Australia (9), Canada (3), South Korea (2), the UK (2), Singapore (1), and Peru (1). For three articles, the country of origin was not specified. Eight studies were diagnosis oriented, for instance, on COPD or intoxicated patients [12, 22, 33, 35, 39, 40, 44, 54].

In Supplementary file 3, we provide a detailed overview of all included studies, including information on study design, location, and quality assessment. The table summarizes for each study: author and year of publication, country and city, patient population (e.g., medical, trauma, inclusion/exclusion criteria), brief study summary, study design (e.g., pre–post comparison, comparison with another hospital), definition and collection of outcomes (with particular attention to readmission, revisit, and mortality), proportion of patients admitted and discharged after observation, characteristics of the unit (e.g., stand-alone or integrated, dedicated or shared staff, number of beds, staff–patient ratio, occupancy rate), characteristics of the hospital (e.g., patient volume, available facilities, annual ED visits), and the quality of evidence as assessed using the GRADE approach.

The included studies were divided into three groups, based on common unit characteristics. The first group, “ACE”, includes acute units specifically designed for older patients, the second group “SSU” includes all types of acute observation units < 24 h, and the third group “Acute Medical Unit (AMU)” includes all remaining acute care units. Because of study heterogeneity with respect to periods of observation, patient characteristics and outcome measures, no formal meta-analysis was performed.

Patient-centered outcomes were categorized in effects on hospital- or emergency department length of stay (ED-LOS), mortality, readmissions, and patient satisfaction. Organizational outcomes were grouped in effects on costs, staffing and ED-congestion.

### Patient-centered outcomes

**A. Impact on Length of Stay**

Of the 50 included studies, 38 studies reported on the length of stay. Results are summarized in Table 1. Overall, reductions between 0.24 and 6.16 days were observed. Twenty-nine studies demonstrated a shorter duration, of which fifteen studies showed statistically significant results.

**Table 1:** Impact implementation of ACUs (AMU/SSU/ACE) on either ED- or Hospital Length of Stay (LOS)

Author	Length of Stay			p-value
	Acute Medical Unit availabe	Acute Medical Unit not availabe	ED/Hospital LOS	
Lévesque et al. [8]	0.7 ± 1.07 days	1.3 ± 1.4 days (UHA)	ED	n/a
Conway et al. [9]	6.6 days	7.1 days	Hospital	< 0.05
Moloney et al. [10]	5 days	7 days	Hospital	< 0.05
Moloney et al. [11]	4 days	6 days	Hospital	< 0.05
Li et al. [12]	5.7 days	6.8 days		< 0.05
Van der Linden et al. [13]	Non-admitted: 117 min AAU: 226 min	Non-admitted: 105 min AAU: 225 min	ED	< 0.05 0.865
Juan et al. [32]	All patients: 2.7–4 days Only for COPD patients: 3.4 days	Total: n/a COPD patients: 12.9 days	Hospital	Total: n/a COPD patients: P < 0.05
McNeill et al. [33]	7.72 days (Consultant present)	9.06 days (Consultant absent)	Hospital	< 0.05
Kinnear et al. [34]	2.06 days	2.32 days	Hospital	< 0.05
Conway et al. [35]	5.1 days low risk 3.2 days	5.1 days high risk 5.8 days	Hospital	< 0.05
Musiienko et al. [36]	5 days	4 days	Hospital	0.27
Elder et al. [37]	225 min	181 min	ED	< 0.05
	**Short stay unit available**	**Short stay not available**		
Budde et al. [14]	4 h 54 min	8 h 11 min	ED	n/a
Strøm et al. [16]	25 h	93 h	Hospital	< 0.05
Plamann et al. [20]	26.8 h	40 h	Hospital	n/a
Nahab et al. [24]	27 h	64.8 h	Hospital	< 0.05
Perry M et al. [25]	EDOU 17.9 h (11.1–23.9) HMSOU 27.9 h (20.1–43.1)	35.5 h (21.5–62.0)	ED	n/a
Candelli M et al. [38]	5.4 days	10.6 days	Hospital	< 0.05
Russell PT et al. [39]	RSI 0.79	RSI 1.34	Hospital	n/a
Yong et al. [40]	SSU LOS < 72 h: 1.2 ± 0.8 SSU LOS > 72 h: 8.7 ± 26.2	LSU LOS < 72 h: 1.8 ± 0.8 LSU LOS > 72 h 13.1 ± 15.2	Hospital	n/a
Cheng et al. [41]	ED A 192 min ED B 182 min	ED A 179 min ED B 182 min	ED	0.55
Mong et al. [42]	19 h	3 days	Hospital	n/a
Binding et al. [43]	28.7 h	7.9 h	Hospital	n/a
Moon et al. [44]	short-stay ED 6.8 ± 10.0 days short stay other 9.0 ± 12.9 days	General Ward 13.9 ± 22.6 days	Hospital	< 0.05
Decker et al. [45]	median 10.1, mean 12.6 h	median 25.2; mean 50.1 h	Hospital	< 0.05
Ok M et al. [46]	LOS ED 671 min Boarding time 323 min (895)	LOS ED 679 min Boarding time 329 min (957)	ED	0.163 0.237
Downes MA et al. [47]	LOS ED 2.7 h (1.6–4.6)	LOS ED 8.5 h (4.7–14)	ED	< 0.05
Wiler et al. [48]	0–6 h 63% 7–12 h 21% 13–24 h 11% > 24 h 5%	0–6 h 73% 7–12 h 20% 13–24 h 3% > 24 h 4%	ED	n/a
	**Acute Care Elderly available**	**Acute Care Elderly not available**		
Meschi et al. [15]	2012 5.24 days 2013 3.86 days 2014 4.24 days	2012 9.45 days 2013 10.02 days 2014 9.92 days	Hospital	< 0.05 < 0.05 < 0.05
Flood et al. [19]	4.0 days	4.2 days	Hospital	0.34
Norman & Sinha [22]	median 5.9 days mean 8.4 days	median 4.8 days mean 7.3 days	Hospital	n/a
Barnes et al. [26]	6.7 days	7.3 days	Hospital	n/a
Fox et al. [27]	Weighted mean difference − 1.28; 95% CI (− 2.33 to − 0.22)		Hospital	< 0.05
O’Shaugnessy et al. [29]	MD -0.36, 95% CI (-0.99–0.26)		Hospital	I^2^ = 77% (low evidence)
Ribbink et al. [49]	SCU 8.8 days	AGCH 9.9 days	Hospital	0.08
Naouri et al. [50]	11.8 days	13 days	Hospital	n/a
Zelada et al. [51]	7.5 ± 4.3 days	9.92 ± 7.74 days	Hospital	< 0.05
Gruenberg et al. [52]	Median 22.1 h, mean 29.3 h	Median 20.6 h, mean 22.8 h	Hospital	< 0.05

^*^LOS = Length of Stay, UHA = Pre-hospitalization unit, ED = Emergency department, EDOU = Emergency Department Observation Unit, HMSOU = Hospital medical and surgical observation unit, ACE = Acute Care unit for Elderly, AGCH = Acute Geriatric Community Hospital, RSI = Relative Strength Index, SCU = Subacute Care Unit, SSU = Short Stay Unit. For ease of view p values under < 0.05 were not specified

#### AMU

Twelve studies assessed the length of stay (LOS) in an AMU setting. Eight studies reported a shorter LOS for patients managed in an AMU compared to those receiving standard care [8–12, 32–34]. The observed difference in LOS ranged from 0.5 days to 2 days, with seven of these studies showing statistically significant results. One study noted no difference in LOS, which was 5.1 days in both units [35]. Three studies found an increase in LOS, two of which were related to the ED, with a difference of 12 to 44 min; both of these results were statistically significant. The third study showing an increase in LOS was not statistically significant [13, 36, 37].

#### SSU

Sixteen studies examined length of stay in a Short Stay Unit [14, 16, 20, 24, 25, 38–48]. Thirteen studies reported a reduction in LOS, with six of these showing statistically significant reductions [16, 24, 38, 44, 45, 47]. The decrease in LOS ranged from 0.24 days to 5.2 days. Three studies noted an increase in LOS, ranging from 13 min to 20.8 h in the Emergency Department. Half of the sixteen studies focused on LOS in the ED, while the other half assessed LOS in the hospital.

#### ACE

Ten out of the twelve ACE studies examined length of stay. Eight of these reported a decrease in LOS [15, 19, 26, 27, 29, 49–51], with reductions ranging from 1.1 to 6.16 days. Only three of these studies found statistically significant results [26, 27, 51]. Two studies noted an increase in LOS, ranging from 6.5 h to 1.1 days. The 6.5-h increase was observed in the Emergency Department and was statistically significant [22, 52]. This was the only study to examine LOS in the ED, while the other nine studies focused on LOS in the hospital.

**B. impact on readmissions**

Of the 50 included studies, 22 studies (40%) reported readmission rates which are shown in Table 2. Eighteen studies reported either no difference (2 studies) or a decrease (17 studies) in readmission rates when an acute unit was implemented. Note: one study reported results for multiple years, contributing to more than one outcome [15]. Among the studies, a decrease in readmissions was observed in 7 out of 12 ACE studies, with two of these findings being statistically significant. Strom C et al. reported the largest reduction in readmissions, noting a 16% decrease following the implementation of an SSU [23].

**Table 2:** Impact of Acute Care Units (AMU/SSU/ACE) on readmission rates

Author	Readmission		p-value
	Acute Medical Unit availabe	Acute Medical Unit not availabe	
Moloney et al. [10]	No change	No change	n/a
Li et al. [12]	7 days: 3.7% 28 days: 8%	7 days: 3.8% 28 days: 8.7%	0.8
Juan et al. [32]	Total 3,1–6.1% COPD: 9.9%	Total: n/a COPD: 7%	COPD = < 0.05
McNeill et al. [33]	30 days: 10.5% 60 days: 18.9%	30 days: 10.2% 60 days: 20.3%	1
Kinnear et al. [34]	30-day ED representation rate: 5.2%	30-day ED representation rate: 5.5%	0.657
Conway et al. [35]	10.5%	10.5%	n/a
Musiienko et al. [36]	7.7%	7.0%	0.24
	**Short Stay Unit available**	**Short Stay Unit not available**	
Strøm et al. [16]	27%	35%	0.28
Candelli M et al. [38]	2%	3%	1
Russell PT et al. [39]	RSI 0.96 (7 days) RSI 0.85 (28 days)		28 day < 0.05
Cheng et al. [41]	ED A 17% ED B 18.3% ED A Non-OU = 14.2% OU Admits = 13.9% ED B Non-OU = 7.5% OU Admits = 8.2%	ED A 17.8% ED B 18.9% ED A 12.3% ED B 7.7%	0.09
Decker et al. [45]	ED-revisits: 33%	ED-revisits: 35%	> 0.05
Bradas C et al. [53]	17.3%	25.8%	n/a
Strom C et al. [54]	30 days: 12.9%	30 days: 28.9%	< 0.05
	**Acute Care Elderly available**	**Acute Care Elderly not available**	
Meschi et al. [15]	2012 11.6% 2013 14.5% 2014 13.3%	2012 13.3% 2013 12.3% 2014 12.5%	0.111 < 0.05 0.293
Flood et al. [19]	7.9%	12.8%	< 0.05
Barnes et al. [26]	3 months: 20%	3 months: 19%	> 0.05
Fox et al. [27]	RR 1.05 (0.92–1.18)	RR 0.69	0.49
O’Shaugnessy et al. [29]	30 days: RR 1.01, 95% CI (0.80–1.28)		I^2^ = 53% (low certainty evidence)
Ribbink et al. [49]	Admission to acute hospital SCU 2.4%	Acute Geriatric Community Hospital 5.2%	< 0.05
Naouri et al. [50]	1 month: 3.5%	1 months: 4.3%	> 0.05
Gruenberg et al. [52]	30 days: 15.5%	30 days: 18.5%	0.31

For ease of view p values under < 0.05 were not specified

CI = Confidence Interval, RSI = Relative Strength Index The **Relative Stay Index (RSI)** is a standardized measure of hospital efficiency, particularly useful when comparing length of stay (LOS) across patient groups adjusting for factors such as age, diagnosis-related group, illness complexity, and institutional case mix. An RSI value **greater than 1.0** indicates less efficient performance than the national average, while an RSI **below 1.0** signals better-than-average efficiency, RR = Relative Risk

#### AMU

Among the 15 carried out in AMUs, seven studies examined readmission rates [10, 12, 32–36]. Of these, two studies reported no difference in readmission rates, three studies observed a small decrease and two studies noted an increase in readmissions, with one of these finding a statistically significant result for COPD patients [32]. They reported an increase in readmissions of 0.7 to 2.9% for AMUs compared to general wards. McNeill et al. found a slight increase of 0.3% in readmissions within the first 30 days, but after 60 days, they observed a decrease of 1.4% when comparing the AMU to a general ward [33].

#### SSU

Among the 23 studies carried out in SSUs, seven studies reported a shorter readmission rate compared to standard care, with only two studies showing a statistically significant difference [16, 23, 38, 39, 41, 45, 53]. One of these studies, conducted by Strom C et al., found that there was a 16% decrease in readmissions within 30 days following the introduction of an SSU [23]. This study was conducted in Denmark as a single-center randomized controlled trial, comparing the SSU with the internal medicine department, with 215 patients in each group.

#### ACE

Among the 12 studies carried out in ACE units, eight analysed readmission rates [15, 19, 26, 27, 29, 49, 50, 52]. Among these, seven studies reported a decrease in readmissions after implementing an ACE unit, with two of these findings being statistically significant [19, 49]. Two studies observed an increase in readmission rates, including one study with a statistically significant result. Note: Meschi et al. reported results for three separate years, with non-significant decreases in readmissions for two years and a statistically significant increase in readmissions during the middle year (2013), contributing to more than one outcome [15].

**C. Impact on mortality**

Mortality rates following the implementation of new acute care units were analysed in 22 of 50 studies. Ten of these studies focused on in-hospital mortality, and found a decrease in mortality ranging from 0.4 to 3%. Other studies examined mortality in the ED (1), 30-day in-hospital mortality (5), 90-day mortality (3), and three studies did not specify the examination period of mortality. Of these 22 studies, eight reported a statistically significant reduction on mortality (Table 3).

**Table 3:** Impact of Acute Care Units (AMU/SSU/ACE) on mortality rates

Author	Mortality (%)		p-value
	Acute Medical Unit available	Acute Medical Unit not available	
Conway et al. [9]	4.7	7	< 0.05
Moloney et al. [10]	1 year: 10.8	1 year: 12.6	0.07
Li et al. [12]	3.7	4.6	n/a
Strøm et al. [23]	90 days: RR 0.73 95% CI (- 0.47; 0.15)		I2 = 0%, very low‐certainty evidence
Juan et al. [32]	COPD patients 1.7	COPD patients:8.1	< 0.05
McNeill et al. [33]	9.4	10.1	0.55
Kinnear et al. [34]	0.5	0.6	0.822
Conway et al. [35]	30 days: 4.6	30 days: 7	< 0.05
Musiienko et al. [36]	Overall: 1.2	Overall: 6.3	< 0.05
Elder et al. [37]	Overall: 1	Overall: 0.5	n/a
	**Short Stay Unit available**	**Short Stay Unit not available**	
Strøm et al. [16]	90 days: 21	90 days: 27	0.36
Candelli M et al. [38]	0	3	0.76
Russell PT et al. [39]	0.48 (incident rate ratios)	1	< 0.05
Moon et al. [44]	short-stay ED 1.9 short stay other 2.2	General Ward 4.1	< 0.05
Ok M et al. [46]	30 days: 2.1	30 days: 2.7	0.292
Strom C et al. [54]	90 days: 10.6	90 days: 15.2	OR 0.66, p = 0.16
	**Acute Care Elderly available**	**Acute Care Elderly not available**	
Meschi et al. [15]	2012 9 2013 8 2014 6	2012 8 2013 8 2014 8	0.941 0.878 < 0.05
Flood et al. [19]	1.4	1.8	> 0.05
Norman & Sinha. [22]	5.4	6.9	0.11
Fox et al. [27]	1.01 (0.81–1.27)		0.9
O’Shaugnessy et al. [29]	RR 0.89, 95% 0.68–1.17		I^2^ = 4% (low certainty evidence)

For ease of view p values under < 0.05 were not specified. Mortality rates are in hospital if not stated otherwise

#### AMU

Of the studies on AMUs, ten assessed mortality, with nine reporting a reduction [9, 10, 12, 23, 32–36]. Four of these studies found a statistically significant decrease. The most notable finding was a reduction from 8.1% to 1.7% for COPD patients [32]. This retrospective study, conducted in Spain, analysed data from over 5000 patients. The SSU in question operated annually from November to March. In contrast, the one study that found an increase in mortality reported 1% for the AMU group compared to 0.5% for the non-AMU group, with no p-value provided [37].

#### SSU

Of the studies on SSUs, six assessed mortality, all six studies examining mortality reported a decrease in mortality rates compared to standard care [16, 23, 38, 39, 44, 46]. Of these, two studies found statistically significant reductions, including one by Russell et al., which observed a reduction of more than 50% in mortality rates following the implementation of an SSU (39,44). This retrospective study, conducted in Australia, analysed 23,790 patients, of whom 10,764 were admitted to the SSU. It compared outcomes between general ward care and SSU care.

#### ACE

Of the studies on ACEs, five assessed mortality, all five studies reported either no difference or a reduction in mortality [15, 19, 22, 27, 29]. Meschi et al., who conducted a three-year study, was the only one to find a statistically significant difference, observed in the final year of the study [15]. This study, conducted in Italy, compared the "come and go" ward with other units. The "come and go" ward had 6,580 admissions, while the other wards had 16,546 admissions.

**D. Impact on patient satisfaction**

Five studies assessed patient satisfaction following the implementation of AMUs, SSUs, and ACE units. All studies consistently reported high levels of satisfaction, with percentages ranging from 89 to 100% in various settings.

Arendts et al. found that in an Enhanced Short Stay Unit (ESSU), 89% of patients rated their care as “very good” or “good” [55]. Similarly, Juan et al. reported that 98% of patients described their AMU stay as “good” or “very good” [32]. Plamann et al. observed an impressive increase in willingness to recommend the SSU, rising from 70.4% to a perfect 100%, underscoring strong patient and family endorsement of the care provided [20].

In further support, Binding et al. highlighted that patients with sickle cell disease expressed high satisfaction with a short-stay model designed for managing uncomplicated crises outside the ED, demonstrating that specialized units can effectively meet specific patient needs [43]. O'Shaughnessy et al. found higher satisfaction rates in an Acute Geriatric Unit (AGU) compared to traditional care units, reinforcing that acute units can enhance patient satisfaction across different patient demographics [29].

### Organizational outcomes

**A. Impact on costs**

Ten out of fifty studies reported on costs. Nine studies (90%) reported a decrease in costs when an ACU was implemented [11, 19, 20, 23–27, 29], of which three studies reported a statistically significant decrease. Two out of these three statistically significant cost reductions were seen. Results are summarized in Table 4. Reductions ranged from €162 per patient to nearly €2 million in total.

**Table 4:** Impact of ACUs (AMU/ SSU/ACE) on healthcare costs

Author	Costs		p-value
	Acute Medical Unit available	Acute Medical Unit not available	
Moloney et al. [11]	€1.714.152 Saved		< 0.05
Strøm et al. [23]	RR 0.8, 95% CI (0.54 to 1.19)		I2 = 57%,
	SSU available	SSU not available	
Plamann et al. [20]	10.4% reduction		n/a
Nahab et al. [24]	$2092 Cost difference $1643; 95% CI(1047–2238)	$4922	> 0.05
Perry M et al. [25]	$1279	$2022	n/a
	ACE available	ACE not available	
Flood et al. [19]	$2109 (Total variable direct cost per patient) $5253 total cost per patient Daily cost per patient = $1377	$2480 (Total variable direct cost per patient) $6321 total cost per patient Daily cost per patient = $1539	< 0.05 < 0.05 < 0.05
Barnes et al. [26]	$9,477	$10,451	< 0.05
Fox et al. [27]	Weighted mean difference = − $431.37		0.09
Tanajewski et al. [28]	total cost: $4475 total cost- adjusted: $4412	total cost: $4057 total cost- adjusted: $4110	> 0.05 95% CI: -809, 1156
O’Shaugnessy et al. [29]	MD -$123.79, 95% CI -$567.80 to $320.22)		I^2^ = 45%

For ease of view p values under < 0.05 were not specified

#### AMU

When examining cost reduction, two studies specifically reported statistically significant cost savings associated with AMU care. Moloney et al., demonstrated a reduction of nearly €2 million in a study conducted in Dublin, Ireland. Patients were admitted to one of two reconfigured wards designated as acute medical assessment units (AMAUs), resulting in savings and a reduction in bed occupancy by approximately 4,039 bed-days [11, 23].

#### SSU

Among the 23 studies focused on Short Stay Units (SSU), three studies reported costs, all three of them noted a decrease in costs, ranging from $379.20 to $1643 per patient (20,24,25). None of these studies reported a statistically significant difference. All three studies were conducted in the USA, each with a different study design.

#### ACE

Among the ACE studies, five out of twelve reported on costs, with four reporting reductions and two demonstrating statistically significant savings. Barnes reported a cost reduction of $974 per patient in a study conducted in the USA. This randomized controlled trial (RCT) compared an ACE unit with interdisciplinary teams to standard care, showing that the cost savings were achieved while maintaining patients' functional abilities [19, 26, 27, 29].

Tanajewski observed an increase in costs following the implementation of an ACE unit, although this result was not statistically significant [28]. Overall, ACE units demonstrated the highest consistency in cost reductions among the models reviewed.

**B. Impact on Emergency Department congestion**

Five studies found a decrease in ED congestion, with patient transfers to other hospitals due to congestion dropping from 10.42% to 6.35% [13], boarding times reduced by 10.4% [20], and waiting times for over 8 h statistically significantly decreasing from 28.7% to 1.9% [12]. Additionally, the proportion of patients leaving without being seen decreased by 2.8% [37].

**C. Impact on staffing**

Thirty studies discussed staffing models in acute care settings, reporting various staffing types and their potential influence on patient or organizational outcomes [8, 9, 12, 13, 15, 20, 22, 25, 32–34, 36, 37, 39–41, 43–49, 51, 53–58]. Staffing levels and specialization were key factors, with limited senior staff on weekends linked to longer lengths of stay. Results are summarized in Table 5.

**Table 5:** Potential impact of staffing models on patient- or organizational outcomes

Authors	Type of study	Staff	Outcome
Li et al. [12]	Retrospective study	Acute Assessment Unit (AAU) with bi-daily reviews by the attending physician	Reduced LOS and increased patient flow
Perry et al. [25]	Retrospective study	Emergency Department Observation Units (EDOUs) managed by emergency physicians compared to HMSOU (internal medicine physicians) and NOUs (general hospital services)	Statistically significant reduction in LOS, costs, and admissions
Juan et al. [32]	Retrospective study	Continuously present physicians and specialists in Emergency Department Short Stay Unit	Reduced LOS and mortality, particularly for high-risk patients (e.g., COPD)
McNeill et al. [33]	Retrospective study	Consultant present at least four days per week	Increased direct discharges, reduced LOS
Musiienko et al. [36]	Retrospective study	Acute Surgery Unit with a dedicated emergency consultant surgeon	Reduced mortality, faster surgical decisions and interventions
Elder et al. [37]	Retrospective study	Senior physicians, interns, and 51 full-time nurses	Improved national target compliance, reduced LOS
Russell et al. [39]	Retrospective study	Short Stay Unit staffed by rotating general internists and junior doctors	Reduced mortality and improved patient flow
Yong et al. [40]	Retrospective study	Short Stay Unit (SSU) with limited senior staff availability	Increased LOS during weekends
Cheng et al. [41]	Before-after study	Observation Unit (OU) with ED physicians and ER nurses	Reduced LOS
Binding et al. [43]	Before-after study	Fast-track model with internists, nurses, and pharmacists	Improved pain management, increased discharge rates
Moon et al. [44]	Retrospective study	Emergency Short Stay Ward (ESSW) with supervised first- and fourth-year ED physicians	Reduced LOS and mortality
Realdi et al. [56]	Retrospective study	Rapid Intensive Observation (RIO) Unit with internists and dedicated nursing staff	Reduced LOS (65% discharged within 72 h)
Strom C et al. [54]	RCT	Dedicated ED staff in a Short Stay Unit (SSU)	Reduced adverse events and functional decline in elderly patients

## Discussion

In this systematic review, we evaluated the impact of ACUs on several outcome domains, including length of stay, mortality, costs and readmission rates. By summarizing these findings from studies conducted worldwide, we provide a comprehensive overview of the benefits and challenges associated with various types of ACUs, such as AMUs, SSUs and ACEs.

The main challenge in studying different acute care models is the difficulty of making fair comparisons due to their heterogeneity. Acute units vary widely in design, patient populations, intended length of stay, and staffing. In addition, healthcare systems differ substantially in how acute care is organized, limiting the possibility of drawing universal conclusions. Evidence from Africa and other emerging economies is largely absent, while most available studies come from large teaching or referral hospitals and are predominantly retrospective or pre–post interventional, providing only a moderate level of evidence.

Evidence from English hospitals shows that no two AMUs are configured alike, with wide variation in consultant presence, staffing skill mix, and patient pathways, despite largely similar case mixes. This heterogeneity makes it difficult to evaluate the efficacy of “the” AMU as a uniform intervention. Future studies should therefore focus on identifying which core components of AMU care consistently drive improved outcomes, while encouraging more standardized reporting of unit characteristics to allow meaningful comparison across settings [59]. While looking at an international level each healthcare system has unique characteristics that influence the outcomes of acute care models. For instance, some systems might have robust primary care networks that handle many conditions outside of the hospital setting, while others direct nearly all cases to emergency wards. Nevertheless, several important conclusions can be drawn while leaving room for context-specific approaches.

First, implementation of ACUs reduces length of stay without an increase in readmission rates or mortality. This holds true for time spent in the emergency department as for total admission time. Reducing length of stay is crucial, as it enhances patient throughput and essentially alleviates ED overcrowding. The latter is particularly critical in systems with high patient volumes and overcrowding is associated with adverse patient outcomes [60, 61]. For example, 97% of Emergency Physicians in the UK reported patient boarding times exceeding 24 h due to a shortage of available hospital beds, resulting in patients waiting in corridors until beds become available [60, 61]. Given the aging population worldwide and increasing healthcare demand, these issues are expected to emerge in the near future and warrant new strategies, such as the implementation of ACUs, to guarantee accessible and high-quality acute care [62].

Second, acute units can reduce health care costs. In our review, eight studies highlighted a statistically significant reduction in costs following the implementation of ACUs. This effect is partly explained by a reduction in the length of stay and the efficient use of staff. Given the ongoing financial challenges in healthcare, such as the €300–420 million shortfalls for Dutch hospitals in 2023–2024, and the negative operational margins of 39% of the U.S. hospitals [63, 64] these findings become more and more relevant. However, these ACUs require substantial initial investments, including infrastructure, specialized staff and training. This might pose barriers to implementation in resource-limited settings. To ensure financial sustainability, collaboration with policymakers and insurers is necessary, alongside regular cost–benefit analyses. Interpretation of cost outcomes across ACU studies should be approached with caution, given the wide variation in methods used to calculate economic impact. Many studies relied on crude estimations, such as multiplying reductions in length of stay by average cost per inpatient day, without capturing the true operational costs of acute care units. This approach overlooks key drivers of expenditure, including higher staffing intensity, multidisciplinary team input, and infrastructure needs, which may increase unit costs despite shorter admissions. Furthermore, cost structures differ substantially between healthcare systems, making comparisons across jurisdictions problematic. Consequently, while reported reductions in length of stay suggest potential for efficiency gains, these findings cannot be directly translated into universal cost savings. More rigorous economic evaluations, incorporating both direct and indirect costs, are needed to assess the true financial impact of ACUs.

Third, the implementation of an ACU did not appear to influence mortality. A total of 22 studies examined mortality rates, with 8 reporting a statistically significant decrease, while none found an increase. These findings suggest that the introduction of an ACU may even have a positive impact on mortality, indicating potential benefits in patient outcomes.

Fourth, there are only five studies that have assessed patient satisfaction following the implementation of AMUs, SSUs, and ACEs, and all reported high satisfaction levels. In further support, Binding et al. highlighted that patients with sickle cell disease expressed high satisfaction with a short-stay model designed for managing uncomplicated crises outside the ED, demonstrating that specialized units can effectively meet specific patient needs (43). O'Shaughnessy et al. found higher satisfaction rates in an Acute Geriatric Unit compared to traditional care units, reinforcing that ACUs can enhance patient satisfaction across different patient demographics [29]. Additionally, five studies found statistically significant reductions in ED congestion following the introduction of these specialized units. Patient transfers to other hospitals dropped from 10.42% to 6.35%, while boarding times were reduced by 10.4%. Furthermore, waiting times decreased, with patients waiting over 8 h dropping from 28.7% to 1.9%. These findings suggest that the implementation of specialized units not only improves patient satisfaction but also enhances the operational efficiency of the ED, highlighting their dual benefits in both patient experience and overall ED performance.

A total of 30 studies examined the role of staffing models in influencing patient outcomes across various acute care settings. In SSUs with emergency physicians and trained nurses, improvements in ED throughput and patient flow were noted, highlighting the importance of emergency-specific expertise. ACE units staffed with geriatric professionals showed higher discharge rates to home and lower hospital mortality, emphasizing the effectiveness of specialized care. Additionally, the availability of senior staff was associated with shorter lengths of stay, especially in SSUs, suggesting the value of continuous, experienced staffing. Overall, generalist-oriented physicians with experience in acute care were linked to better outcomes, reinforcing the need for adequate staffing to optimize care in acute units. Although heterogeneous, most units employ similar staffing models of internists and emergency physicians, suggesting opportunities for shared standards and collaboration.

It should also be acknowledged that much of the published evidence on Acute Medical Units (AMUs) originates from a small number of centers, with St James’s Hospital in Dublin being particularly influential. Several papers from this group are frequently cited, and while they provide valuable long-term insights, some report overlapping or identical data (e.g., outcomes at 5, 10, and 15 years). This concentration of evidence may overrepresent the experience of a single institution and limit the generalizability of findings. Future research should therefore aim to broaden the evidence base by incorporating data from diverse healthcare systems and patient populations.

Finally, ACUs may have some limitations. Not all studies showed statistically significant improvement on the set endpoints. Limitations mentioned are often connected to the difficulties of the transdisciplinary design of such units. Studies on ACE units provide relatively higher levels of evidence, which are units particularly for geriatric populations. These units demonstrate the benefits of multidisciplinary teams and rapid assessment, which support early discharge and help preserve functional status in this vulnerable group. By contrast, SSUs and AMUs are highly heterogeneous in design and scope, yet they share the common function of acting as ED-associated filter units for medical patients, aiming to reduce crowding and streamline hospital admissions.

When used as a temporary solution to decrease ED crowding or hospital bed shortage acute care units benefit from a clear infrastructure and protocols. For instance, the maximum length of stay on an acute care unit and clear agreement on responsibility. Acute care units rely on rapid triage and short-term care models, requiring healthcare professionals to quickly switch between different care types. This can lead to increased workload, particularly in the absence of adequate staffing or training, potentially impacting long-term job satisfaction. Ensuring sustainable staff support through ongoing education, appropriate staffing ratios, and well-structured workflows is essential to maximize the benefits of ACUs without compromising staff well-being. A successful acute unit requires an efficient setup that optimally supports collaboration with other departments. This includes flexible spaces and digital communication systems that facilitate swift information exchange, as well as streamlined triage and patient flow protocols.

## Conclusion

Acute Care Units offer transformative potential for modern healthcare systems and may contribute to the accessibility of emergency care without compromising quality. Most benefit is expected when the chosen ACU model, with a matching staffing model, is in line with specific bottlenecks in the local or regional acute care chain to maximize their impact. Future studies should aim to establish a standardized framework for evaluating acute care units, enabling more consistent comparison across settings and models. In addition, higher-quality research designs, including prospective and multicenter studies, are needed to generate more robust evidence on their effectiveness and impact.

## Supplementary Information

Below is the link to the electronic supplementary material.

Supplementary file1 (DOCX 12 KB)

Supplementary file2 (DOCX 58 KB)

Supplementary file4 (DOCX 2615 KB)

## Abbreviations

AAU: Acute assessment unit
ACU: Acute care unit
ACE: Acute care for elderly unit
AGCH: Acute geriatric community hospital
AGU: Acute geriatric unit
AMAU: Acute medical assessment unit
AMU: Acute medical unit
CI: Confidence interval
ED: Emergency department
EDOU: Emergency department observation unit
ESSU: Enhanced short stay unit
ESSW: Emergency short stay ward
HMSOU: Hospital medical and surgical observation unit
LOS: Length of stay
OU: Observation unit
PRISMA: Preferred reporting items for systematic reviews and meta-analyses
RCT: Randomized controlled trial
RIO: Rapid intensive observation
RR: Relative risk
RSI: Relative strength index
SCU: Subacute care unit
SSU: Short Stay unit
UK: United Kingdom
USA: United States of America
