# Supplementary material for: Optimizing efficiency in the acute care chain: a systematic review on the implementation and impact of interdisciplinary short-term monitoring in acute care units
Source: Intern Emerg Med. 2025 Nov 20;21(1):283–95. doi: 10.1007/s11739-025-04194-w (PMC12948887; doi:10.1007/s11739-025-04194-w)
Supplement: Supplementary file 1 — Supplementary file1 (DOCX 12 KB) [file 11739_2025_4194_MOESM1_ESM.docx]

Search string Figure 1 (SI) Search terms

| **(((((((((("Acute Medical Admission Unit") OR ("Acute Medical Assessment")) OR ("Acute Medical Unit")) OR ("Short stay")) OR ("Emergency admissions units")) OR ("Acute Admission Unit")) OR ("Acute Care for Elders")) OR ("Observational units")) OR ("Observation unit")) OR ("Observation ward")) AND ((((((((((((((("Length of Stay"[Mesh]) OR ("Length of Stay")) OR ("Patient Readmission"[Mesh])) OR ("Patient Readmission")) OR ("Mortality"[Mesh])) OR (Mortality[tiab])) OR ("hospital mortality")) OR ("Costs and Cost Analysis"[Mesh])) OR (Costs[tiab])) OR ("Efficiency"[Mesh])) OR (Effectivity[tiab])) OR (benefit[tiab])) OR (profit[tiab])) OR (utility[tiab])) OR (advantage[tiab]))** *Filters:* ***Adult: 19+ years, Young Adult: 19-24 years, Adult: 19-44 years, Middle Aged + Aged: 45+ years, Middle Aged: 45-64 years, Aged: 65+ years, 80 and over: 80+ years, from 2005 - 2024*** |
| --- |
