# Supplementary material for: Optimizing efficiency in the acute care chain: a systematic review on the implementation and impact of interdisciplinary short-term monitoring in acute care units
Source: Intern Emerg Med. 2025 Nov 20;21(1):283–95. doi: 10.1007/s11739-025-04194-w (PMC12948887; doi:10.1007/s11739-025-04194-w)
Supplement: Supplementary file 2 — Supplementary file2 (DOCX 58 KB) [file 11739_2025_4194_MOESM2_ESM.docx]

SUPPLEMENTARY MATERIAL

Table 1 (SI) Explanatory box with commonly used terms and their corresponding definitions as used in this review.

| Acute Care Unit  **(ACU)** | Overarching term for departments focused on the acute admission and treatment of patients. |
| --- | --- |
| Acute Observation Area  **(AOA)** | Provides short-term, closely monitored care for patients needing urgent treatment and observation before being admitted or discharged, usually <72 hours. |
| Acute Medical Unit  **(AMU)** | Provides short-term, closely monitored care for medical patients needing urgent treatment and observation before being admitted or discharged, usually <72 hours. |
| Short Stay Unit  **(SSU)** | Provides short-term, closely monitored care for patients needing urgent treatment and observation before being admitted or discharged, usually <24 hours. |
| Acute Care for Elders  **(ACE)** | Provides short-term, closely monitored care for older patients (65+) needing urgent treatment and observation before being admitted or discharged, usually <72 hours. |
| General Ward **(GW)** | A standard inpatient hospital ward where patients receive medical care. |
| Emergency Department  **(ED)** | Medical facility in hospitals that provides immediate and acute care for patients with urgent or life-threatening conditions. |
| Emergency Short Stay Ward  **(ESSW)** | Provides short-term, closely monitored care for patients needing urgent treatment and observation before being admitted or discharged, usually 24-48 hours, often attached to the ED. |
| Acute Assessment Unit  **(AAU)** | Provides short-term, closely monitored care for patients needing urgent treatment and observation before being admitted or discharged, usually <72 hours. |
| Emergency Department Short Stay Unit  **(EDSSU)** | Provides short-term, closely monitored care for patients needing urgent treatment and observation before being admitted or discharged, usually <24 hours, often attached to the ED. |
| Emergency Department Observation Unit **(EDOU)** | Provides short-term, closely monitored care for patients in the Emergency Department who require extended evaluation and treatment before being admitted or discharged, usually <24 hours. |
| High-Medical Supervision Observation Unit **(HMSOU)** | Provides intensive, closely monitored care for patients requiring a higher level of medical supervision than standard observation units but not full intensive care. |
| Emergency medicine **(EM)** | Branch of medicine which is specialized for medical emergency requiring immediate response, including initial resuscitation and stabilization in patients in EDs. |
| Acute Geriatric Community Hospital **(AGCH)** | Provides specialized, short-term, closely monitored care for older patients (typically 65+) requiring acute medical treatment and rehabilitation in a community hospital setting, often as an alternative to prolonged hospital admission. |
| Long-stay unit **(LSU)** | LOS>72 h |
| Rapid Triage | A process in which patients are quickly assessed based on the severity of their condition to ensure they receive the appropriate care as soon as possible. This is a core process in ACUs due to the short care duration and high patient turnover. |
| Patient Boarding | A situation where patients, due to a lack of available hospital beds, have to wait for extended periods in the Emergency Department or are temporarily placed in corridors. |
| Staff-to-Patient Ratio | The number of healthcare providers per patient in a specific department or care setting. |
| Cost-Benefit Analysis | An economic evaluation of the costs and benefits of a healthcare intervention, such as an ACU, to determine if the benefits justify the costs. |
|  |  |

Search string Figure 1 (SI) Search terms

| **(((((((((("Acute Medical Admission Unit") OR ("Acute Medical Assessment")) OR ("Acute Medical Unit")) OR ("Short stay")) OR ("Emergency admissions units")) OR ("Acute Admission Unit")) OR ("Acute Care for Elders")) OR ("Observational units")) OR ("Observation unit")) OR ("Observation ward")) AND ((((((((((((((("Length of Stay"[Mesh]) OR ("Length of Stay")) OR ("Patient Readmission"[Mesh])) OR ("Patient Readmission")) OR ("Mortality"[Mesh])) OR (Mortality[tiab])) OR ("hospital mortality")) OR ("Costs and Cost Analysis"[Mesh])) OR (Costs[tiab])) OR ("Efficiency"[Mesh])) OR (Effectivity[tiab])) OR (benefit[tiab])) OR (profit[tiab])) OR (utility[tiab])) OR (advantage[tiab]))** *Filters:* ***Adult: 19+ years, Young Adult: 19-24 years, Adult: 19-44 years, Middle Aged + Aged: 45+ years, Middle Aged: 45-64 years, Aged: 65+ years, 80 and over: 80+ years, from 2005 - 2024*** |
| --- |

Table 2 (SI)

***ACE***

| **Author** | **Length of Stay** | | **p-value** | **Mortality** | | **p-value** | **Costs** | | **p-value** | **Readmissions** | | **p-value** | **Other results** | **ref** |
| --- | --- | --- | --- | --- | --- | --- | --- | --- | --- | --- | --- | --- | --- | --- |
|  | **Amu** | **Non-amu** |  | **Amu** | **Non-amu** |  | **Amu** | **Non-amu** |  | **Amu** | **Non-amu** |  |  |  |
| **Meschi et al.** | 2012 5.24 (days)  2013 3.86  2014 4.24 | 2012 9.45  2013 10.02  2014 9.92 | <0.001  <0.001  <0.001 | 2012 9%  2013 8%  2014 6% | 2012 8%  2013 8%  2014 8% | 0.941  0.878  0.018 | n/a | n/a | n/a | 2012 11.6%  2013 14.5%  2014 13.3% | 2012 13.3%  2013 12.3%  2014 12.5% | 0.111  0.008  0.293 | the IM and come’n’go ward of Internal Medicine and Critical Subacute Care Unit  admissions from ED  97%  other units  81% | 14 |
| **Norman & Sinha** | median 5.9 days  mean 8.4 days | median 4.8 days  mean 7.3 days | n/a | 5.4% | 6.9% | .11 | n/a | n/a | n/a | n/a | n/a | n/a | ACEUnit were both more likely to be discharged home (oddsratio  [OR]  1.31,  95%  confidence  interval  1.12–1.54,p=0.001) and less likely to die in hospital (OR 0.70[0.51–0.95],p=0.02  AFTER CASEMIX  ACEUnit were both more likely to be discharged home (oddsratio  [OR]  1.31,  95%  confidence  interval  1.12–1.54,p=0.001) and less likely to die in hospital (OR 0.70[0.51–0.95],p=0.02 | 18 |
| **Flood et al.** | 4.0 | 4.2 | .34 | 1.4% | 1.8% | n/a | $2109 (Total variable direct cost per patient)    5253 total cost per patient    Daily cost per patient= 1377 | $2480 (Total variable direct cost per patient)    6321 total cost per patient    Daily cost per patient= 1539 | .009    <.001    <.001 | 7.9% | 12.8% | .02 | Adjusted cost ratios revealed statistically significant cost savings for patients with low (0.82; 95% CI, 0.72-0.94) or moderate (0.74; 95% CI, 0.62-0.89) CMI scores; care was cost neutral for patients with high CMI scores (1.13; 95% CI, 0.93-1.37). | 19 |
| **Barnes et al.** | 6.7D | 7.3D | n/a | In-hospital: 5% | In-hospital: 3% | >0.05 | $9,477 | $10,451 | n/a | 3 month: 20% | 3 month: 19% | n/a | n/a | 24 |
| **Fox et al.** | (WMD = −1.28, 95% CI = −2.33 to −0.22; P = .02) |  |  | 1.01 (0.81–1.27) |  | 0.9 | (WMD = −$431.37, 95% CI = −$933.15–$70.41; P = .09) |  |  | 1.05 (0.92–1.18) 0.69 |  | .49 | n/a | 25 |
| **Tanajewski et al.** | n/a | n/a | n/a | n/a | n/a | n/a | total cost: 4475  total cost- adjusted: 4412 | total cost: 4057  total cost- adjusted: 4110 | non sig  95% CI: -809, 1156 | n/a | n/a | n/a | n/a | 26 |
| **O’Shaugnessy et al** | LOS-H  MD -0.36, 95% CI -0.99-0.26) |  | I^2^ = 77% (low evidence) | RR 0.89, 95% 0.68-1.17 |  | I^2^ = 4% (low certainty evidence) | MD -123.79 USD, 95% CI -567.80USD to 320.22 USD) |  | I^2^ = 45% (low certainty evidence) | 30 days  RR 1.01, 95% CI 0.80-1.28) |  | I^2^ = 53% (low certainty evidence) | Functional decline at discharge AGU: RR 0.89, 95% CI 0.75-1.04; I^2^ = 97% very low certainty evidence) | 27 |
| **Naouri et al.** | 11.8 days | 13 days | n/a | n/a | n/a | n/a | n/a | n/a | n/a | 3.5% 1month | 4,3% | n/a | n/a | 48 |
| **Zelada et al.** | 7.5 +- 4.3 days | 9.92+-7.74 days | 0.03 | death during admission was exclusion criteria | death during admission was exclusion criteria | n/a | n/a | n/a | n/a | n/a | n/a | n/a | n/a | 49 |
| **Gruenberg et al.** | median geriatric 22.1 H  mean 29.3 | median non-geriatric 20.6 H  mean 22.8 | < 0.01 | n/a | n/a | n/a | n/a | n/a | n/a | geriatic (30 days)  15.5% | non-geiatric  18.5% | 0.31 | Geriatric staying longer than 24 h (42% vs. 29.1%; p < 0.01) | 50 |
| **Moyet et al.** | 2.7 days  (73.4% LOS <3 days) | n/a | n/a | 6.3% | n/a | n/a | n/a | n/a | n/a | 10.8% | n/a | n/a | n/a | 58 |

***SSU***

| **Author** | **Length of Stay** | | **p-value** | **Mortality** | | **p-value** | **Costs** | | **p-value** | **Readmissions** | | **p-value** | **Other results** | **ref** |
| --- | --- | --- | --- | --- | --- | --- | --- | --- | --- | --- | --- | --- | --- | --- |
|  | **Amu** | **Non-amu** |  | **Amu** | **Non-amu** |  | **Amu** | **Non-amu** |  | **Amu** | **Non-amu** |  |  |  |
| **Strøm  et al.** | 25 hours | 93 hours | <0.001 | 21% | 27% | 0.36 | n/a | n/a | n/a | 27% | 35% | 0.28 | n/a | 12 |
| **Budde et al.** | 4h 54 min | 8h 11min | n/a | n/a | n/a | n/a | n/a | n/a | n/a | n/a | n/a | n/a | Pre-OU period: 78.0% of ED visits resulted in hospital admissions.  Post-OU period: This dropped to 62.9% of ED visits.\  p = 0.0049 | 13 |
| **Plamann et al.** | 26.8H | 40H | n/a | n/a | n/a | n/a | reduced by 10.4% for a direct cost reduction of $379.20 per patient. | n/a | n/a | n/a | n/a | n/a | n/a | 20 |
| **Nahab et al.** | 27H | 64.8H | <.01 | n/a | n/a | n/a | 2092    cost difference  $1643 | 4922 | n/a | n/a | n/a | n/a | median LOS of all post-ADP patients (ADP and non-ADP) was 20.8 hours shorter (95% CI, 16.3-25.1 hours; P < .01) than that of the pre-ADP patients    Post-ADP patients managed using the ADP had a median LOS of 25.5 hours, 37.6 hours shorter than that of non-ADP patients (95% CI, 32.7-42.5 hours; P < .01). | 22 |
| **Perry M et al.** | EDOU 17.9 hours (11.1-23.9)  HMSOU 27.9 hours (20.1-43.1) | 35.5 hours (21.5-62.0) | n/a | na | n/a | n/a | 1269/1671 | 1461 | n/a | n/a | n/a | n/a | Admission rate EDOU/HMSOU/NOU 12.3-15.6-26.4 | 23 |
| **Candelli M et al.** | 5.4 days | 10.6 days | <0.0001 | 0% | 3% | 0.76 | n/a | n/a | n/a | 2% | 3% | 1 | n/a | 35 |
| **Russell PT et al.** | RSI 0.79 | RSI 1.34 |  | 0.48 (incident rate ratio’s) | 1 | < 0.05 | n/a | n/a |  | 0.96(7 days)  0.85 (28 days) | 1 | 28 day< 0.05 | Patients in the SSU who stayed longer than 72 hours had a Relative Stay Index (RSI) of 1.29, indicating less efficient care for this group.  Patients in the LSU who were discharged within 72 hours had an RSI of 0.40, suggesting that the LSU was more efficient in treating short-stay patients than the SSU. | 36 |
|  |  |  |  |  |  |  |  |  |  |  |  |  |  |  |
| **Sherwood et al.** | 14h 32min  chest pain  12h 46min trauma | n/a | n/a | n/a | n/a | n/a | n/a | n/a | n/a | n/a | n/a | n/a | n/a | 37 |
| **Yong et al.** | SSU en LOS <72u:  1.2 ± 0.8  SSU en LOS >72u: 8.7 ± 26.2 | LSU en LOS <72u: 1.8 ± 0.8  LSU en LOS> 72u  13.1 ± 15.2 | n/a | 2% | n/a | n/a | n/a | n/a | n/a | 3% | n/a | n/a | proportion of patients discharged within 72 h was statistically significantly lower when their admissions occurred over the weekend. | 38 |
| **Cheng et al.** | ED A  Post-OU (all)= 192 min  ED B  Post-OU(all)= 182 min    ED A  Post-OU  Non-OU= 190 min  OU Admits= 302 min  ED B  Post-OU  Non-OU= 181 min  OU Admits= 243min | ED A  Pre-OU= 179 min  ED B  Pre-OU= 182 min    ED A  Pre-OU= 179 min  ED B  Pre-OU= 182 min | 0.55 | n/a | n/a | n/a | n/a | n/a | n/a | ED A  (72H) Post-OU (all)= 17%  ED B  Post-OU(all)= 18.3%    ED A  Post-OU  Non-OU= 14.2%  OU Admits= 13.9%  ED B  Post-OU  Non-OU= 7.5%  OU Admits= 8.2% | ED A  Pre-OU= 17.8%  ED B  Pre-OU= 18.9%    ED A  Pre-OU= 12.3%  ED B  Pre-OU= 7.7% | 0.09 | OU   (17.8% vs17.0% P= 0.01  18.9% vs 18.3%   P=0.09) | 39 |
| **Mong et al.** | 19 hours | 3 days | n/a | 0% | n/a | n/a | n/a | n/a | n/a | 3.5% within 2 weeks. 1 patient with persistent pain after a bite. Others were due to relaps depression/admitted to a psychiatric ward. | n/a | n/a | n/a | 40 |
| **Binding et al.** | 28.7 H | 7.9H | n/a | n/a | n/a | n/a | n/a | n/a | n/a | n/a | n/a | n/a | discharge rate amu= 84.2%  ED= 69.7 %  time to first opiate (min) AMU= 23.5M  ED=100.3M  p=<0.001\  Satisfaction with care at the short-stay unit was ranked very highly overall and also with respect to specific aspects of care, with all the average ratings above four out of five on a five-point Likert scale with one exception. | 41 |
| **Moon et al.** | short-stay EM  6.8 ± 10.0 days  short stay other  9.0 ± 12.9 | GW 13.9 ± 22.6 | <0.001 | short-stay EM  156 (1.9%)  short stay other  51 (2.2%) | GW 656 (4.1%) | <0.0001 | n/a | n/a | n/a | n/a | n/a | n/a |  | 42 |
| **Decker et al.** | median 10.1, mean 12.6 hours | median 25.2; mean 50.1 hours | <0.01 | 0 | 0 | n/a | n/a | n/a | n/a | 33% revisits | 35% revisits | Non sig | n/a | 43 |
| **Ok M et al.** | LOS ED  671 minutes  Boarding time  323 minutes (895) | LOS ED  679 minutes  Boarding time  329 minutes (957) | 0.163  0.237 | 30 days  2.1 | 2.7 | 0.292 | n/a | n/a | n/a | n/a | n/a | n/a | n/a | 44 |
| **Downes MA et al.** | LOS ED  Hours  2.7 (1.6-4.6)  LOS-H  16.7 (11.5-23) | LOS ED  Hours  8.5 (4.7-14)  LOS-H  14.5 (8.4-21.8) | <0.0001 | 1 | 0 | n/a | n/a | n/a | n/a | n/a | n/a | n/a | n/a | 45 |
| **Wiler et al.** | Hours  0-6 63%  7-12 21%  13-24 11%  >24 5% | Hours  0-6 73%  7-12 20%  13-24 3%  >24 4% | n/a | n/a | n/a | n/a | n/a | n/a | n/a | n/a | n/a | n/a | n/a | 46 |
| **Strom C et al.** | n/a | n/a |  | 90-day  10.6% | 90-day  15.2% | OR 0.66, p = 0.16 | n/a | n/a |  | 30 day  12.9% | 30 day  28.9% | OR 0.37, p <0.001 |  | 53 |
| **Arendts et al.** | n/a | n/a | n/a | n/a | n/a | n/a | n/a | n/a | n/a | 9% readmitted for the same problem |  |  | 187/211 patients (89%) stated that they received some or substantial benefit from their ESSU stay | 54 |
| **Shetty et al.** | n/a) | n/a | n/a | n/a | n/a | n/a | n/a | n/a | n/a | n/a | n/a | n/a | n/a | 55 |

***AMU/ASU***

| **Author** | **Length of Stay** | | **p-value** | **Mortality** | | **p-value** | **Costs** | | **p-value** | **Readmissions** | | **p-value** | **Other results** | **ref** |
| --- | --- | --- | --- | --- | --- | --- | --- | --- | --- | --- | --- | --- | --- | --- |
|  | **Amu** | **Non-amu** |  | **Amu** | **Non-amu** |  | **Amu** | **Non-amu** |  | **Amu** | **Non-amu** |  |  |  |
| **Lévesque et al.** | 0.7  ±  1.07 days (UHCD) | 1.3  ±  1.4 days (UHA)  (UHA=Pre-hospitalization unit) |  | ? | 3.8% (UHA) |  | n/a | n/a |  | n/a | n/a |  |  | 6 |
| **Conway et al.**  **O’Riodan, silke** | 6.6 days | 7.1 days | < 0.001 | 4.7% | 7% | 0.001 | n/a | n/a |  | n/a | n/a |  |  | 7 |
| **Moloney et al.** | 4 days | 6 days | <0.0001 |  |  |  | €1.714.152 Saved |  |  | n/a | |  |  | 8 |
| **Moloney et al.** | 5 days | 7 days | <0.0001 | 10.8% | 12.6% | 0.07 | n/a | n/a |  | No change | No change |  |  | 9 |
| **Li et al.** | 5.7days | 6.8days | <0.001 | 3.7% | 4.6% |  | n/a | n/a |  | 7 days 3.7%  28 days 8% | 7 days 3.8%  28 days 8.7% | 0.8 | Matched patients in 2006 had a mean LOS of 6.0 days P < 0.001  matched mortality 4.2% | 10 |
| **van der Linden et al.** | Non-admitted= 117min  FAAU= 226 | Non-admitted = 105 min  FAAU= 225 | < .001  .865 | n/a | n/a | n/a | n/a | n/a | n/a | n/a | n/a | n/a | Transfers due to full beds dropped from 10.42% to 6.35% (P = .0037) after intervention. | 11 |
| **Strøm et al.** | did not perform meta‐analyses |  |  | RR 0.73  95% CI - 0.47 to .15 |  | I2 = 0%, very low‐certainty evidence | RR 0.8  95% CI 0.54 to 1.19 |  | I2 = 57%, very low‐certainty evidence | All trials indicated that the short‐stay unit model had cost‐saving properties compared with usual care. |  |  |  | 21 |
| **Juan et al.** | All patients: 2.7-4 days  Only for COPD patients: 3.4 days | Total:  n/a  COPD patients:12.9 days | Total: n/a  COPD patients:P<0.05 | Total: n/a  COPD patients 1.7% | Total: n/a  COPD patients:8.1% | Total: n/a  COPD patients <0.05 | n/a | n/a | n/a | Total 3,1-6.1 %  COPD: 9.9% | Total:  n/a  COPD:  7% | COPD= 0.05 | EDSSU: No LOS impact, 98% patient satisfaction, noise issues. | 30 |
| **McNeill et al.** | Consultant present: 7.72 days | Consultant absent: 9.06 days | 0.048 | Consultant present:  9.4% | Consultant absent:  10.1% | 0.55 | n/a | n/a | n/a | Consultant present:  30 days: 10.5%  60 days: 18.9% | Consultant absent:  30 days: 10.2%  60 days: 20.3% | 1 |  | 31 |
| **Conway et al.**  **bruyne, cournane** | 5.1 days  low risk  3.2 days | 5.1 days  high risk  5.8 days | <0.001 | 4.6%  low risk  0.2% | 7%  high risk  5.7% | 0.001    <0.001 | n/a | n/a | n/a | 18.1% | 2.9% |  |  | 32 |
| **Musiienko et al.** | 5 (IQR7) | 4 (IQR5) | 0.27 | 2 (1.2%) | 9 (6.3%) | 0.02 | n/a | n/a | n/a | 15 (7.7%) | 12 (7.0%) | 0.24 |  | 33 |
| **Elder et al.** | 225min | 181min | <0.001 | 1% | 0.5% | n/a | n/a | n/a | n/a | n/a | n/a | n/a |  | 34 |
| **Kinnear et al.** | 2.32 days | 2.06 days | <0.0001 | 9 (0.5%) | 14 (0.6%) | 0.822 | n/a | n/a | n/a | 88 (5.2%) (30 day ED representation rate) | 132 (5.5%) (30 day ED representation rate) | 0.657 |  | 51 |
| **Realdi et al.** | 65% RIO success = discharge <72u hours. LOS: 2.4±0.7days  35% RIO failures (admitted to AMU but stay >72h) LOS: 7.4±4.1days.  Overall LOS: 4.1±3.4day |  | n/a | N=1 | n/a | n/a | n/a | n/a | n/a | 9.4 ± 3.5 in RIO patients 7.0 ± 3.9 in RIO failure patients | n/a | Not significant | Younger age, high Barthel, low CIRS predict RIO success. | 56 |

| **Brief summary** | **Author** |
| --- | --- |
| randomized to the Acute Care for Elders intervention group versus randomized to the usual-care control group. | **Barnes et al.** |
| ACE patients were assigned to either the ACE unit, designed specifically for their care, or were distributed to other units. | **Norman & Sinha** |
| Short-Stay Unit (SSU) hospitalisation versus internal medicine department (IMD) hospitalisation.  conclusion: Patients aged 75 and older had fewer adverse events in a short stay unit than in an Internal Medicine Department.  Evaluating adverse events. | **Strøm  et al.** |
| Pre-hospitalization unit (UHA) that functions as a buffer during peak hours, 20 places  And an AMU (UHCD) 16 places    conclusion: The waiting hospitalisation unit at Rouen University Hospital improved emergency patient flow, but weekend bed shortages and epidemic management remain challenges. | **Lévesque et al.** |
| the Acute Medical Admissions Unit (AMAU) was established in 2003; now, they are retrospectively reviewing all patients, including those from the Intensive Care Unit (ICU) and High Dependency Unit (HDU), from 2002 to 2012 to assess the differences between 2012 and 2002.  Conclusion: AMAU model resulted in significant long-term reductions in in-hospital mortality, length of stay, and emergency department wait times, | **Conway et al.**  **O’Riodan, silke** |
| Acute medical admissions unit (AMAU) versus various non-specialty wards.  Conclusion: AMAU reduces LOS, waiting time and costs | **Moloney et al.** |
| Acute medical admissions unit (AMAU) versus various non-specialty wards. | **Moloney et al.** |
| The SSU was compared immediately after opening and 12 months later.  Conclusion: After a year, the SSU had lower 30-day readmission rates for all diagnoses, suggesting better patient selection and improved discharge processes. | **Bradas C et al.** |
| The study compares outcomes and resource use between patients in regular wards and those in an SSU.  Conclusion: Treating NVUGIB in the SSU led to shorter hospital stays, quicker endoscopy times, and fewer transfusions. | **Candelli M et al.** |
| Assess the impact of an SSU on the efficiency of the department by comparing SSU versus general ward and LSU (long stay unit)  Conclusion: An SSU for general medical patients is a safe alternative to a traditional ward, but it does not significantly enhance overall efficiency or expedite discharge compared to normal care. | **Russell PT et al.** |
| Comparing general care before a functional AAU and after a functional AAU  Conclusion:The introduction of an Acute Assessment Unit (AAU) led to significant improvements in performance, including shorter hospital stays and an increase in direct discharges, without an increase in hospital mortality or unplanned readmissions | **Li et al.** |
| Compared the total number of hospital admissions before and after the availability of an Observation Unit (OU), as well as the number of patients discharged directly from the Emergency Department (ED).  Conclusion: The introduction of an Observation Unit (OU) for managing AECOPD led to a significant and immediate reduction in COPD hospital admissions while maintaining stable discharge rates from the Emergency Department | **Budde et al.** |
| Examine variable direct costs from an interdisciplinary ACE compared with a multidisciplinary usual care (UC) unit.  Conclusion: The ACE unit team model reduces costs and 30-day readmissions. | **Flood et al.** |
| Seeing if PAs are able to staff the EDOU  Conclusion: PAs effectively cared for patients | **Sherwood et al.** |
| The comparison is between the pre-ADP period, when all patients were admitted to neurology, and the post-ADP period, where patients were managed via the ADP or admitted based on exclusion criteria or physician discretion.  The patients evaluated during the first 11 months of the study (pre-ADP period) were compared with those evaluated in the subsequent 7-month period after the ADP was instituted (post-ADP period). | **Nahab et al.** |
| Implementation of an observation unit to improve patient flow, reduce costs, and enhance patient satisfaction. Pre-implementation of the observation unit vs. Post-implementation  Conclusion: The establishment of an adult observation unit improved care for patients and families through an evidence-based, interdisciplinary approach that enhanced outcomes like LOS and fostered care innovation. | **Plamann et al.** |
| They analyzed data from a 4-month control period in 2008 and a 4-month intervention period in 2009. During the intervention period, an FAAU with 15 inpatient beds was implemented for off hours.  Conclusion: The FAAU reduced ED crowding by cutting patient boarding and preventing longer ED stays. | **van der Linden et al.** |
| Geriatric ( ≥ 65 Years) compared to non-geriatric patients (< 65 Years)  Conclusion:  Geriatric and non-geriatric patients on an EDOU chest pain pathway have similar 30-day re-presentations; geriatric patients experience longer LOS, partly due to more nuclear stress tests. | **Gruenberg et al.** |
| evaluated two hospitals before OU implementation and after OU implementation  compared three groups of patients: The pre-OU cohort, the post-OU cohort, and the latter subcategorized into those who were managed in the OUs (post-OU) and those who were not (post-non-OU).    Conclusion: OU implementation decreased hospital admissions at one site but did not reduce ED LOS for all patients. | **Cheng et al.** |
| inpatient wards versus EDOU (Emergency Department Observation Unit)  Conclusion: EDOU patients are less likely to have a diagnosed etiology than admitted patients. | **Grossman et al.** |
| SCU vs AGCH  Conclusion: acute geriatric units in two settings are similar alternatives to general hospitals. | **Ribbink et al.** |
| ACE versus general care  Conclusion: Acute geriatric care with ACE components significantly reduces falls, delirium, functional decline, hospital stay, and costs, while increasing home discharges, particularly for octogenarians. | **Fox et al.** |
| Comparison before and 15 years after the opening of an AMAU. Meanly comparing high risk patients vs low risk | **Conway et al.**  **bruyne, cournane** |
| Comparing the IM and come’n’go ward of Internal Medicine and Critical Subacute Care Unit with other units  Conclusion: The geriatric care model reduces in-hospital mortality and length of stay, but does not significantly lower 30-day readmission rates. | **Meschi et al.** |
| Patients with ‘intermediate risk’ medical problems where admitted to a rapid intensive observation unit (RIO). With dedicated and fast diagnostic tools. With the goal of discharging <72u hours.  Outcome divided in RIO patients and RIO-failure (admission to normal ward) | **Realdi et al.** |
| To evaluate their ability to predict a patient’s likely length of stay (LOS) And to describe differences between patients who were allocated inappropriately to either the long-stay unit (LSU) (i.e. LOS < 72 h) or to the SSU (LOS>72 h). Finally, evaluation of the quality of care afforded to short-stay patients  the establishment of a medical SSU at FMC allowed accurate identification of a stream of patients, who could be treated safely without compromising patients’ outcomes. The accuracy of prediction of long and short-stay patients improved over time. | **Yong et al.** |
| AMAU since 2003, reviewing data for LOS, mortality rate between 2002-2017  Results: improving mortalityrate over 16 years period, unaltered re-admission and LOS. No comparison with a normal ward. | **Conway et al.** |
| Goal: to describe the management of patients with toxic exposure in a short stay obersvation unit.  Conclusion: Most patients (93%) admitted to the EDOU (emergency department observation unit) were successfully managed and medically cleared within 23 hours, including those with a severe PSS. (poison severity score due to a GCS 8 or less). Previous studies showed a local LOS on a normal ward of 3 days. Admission on a AMU/EDOU could decrease the LOS for patients with toxic exposure | **Mong et al.** |
| a trauma protocol was implemented for the ED observation unit.  364 trauma patients were admitted to the observation unit. 84.6% were trauma II activations and 3.8% were trauma I activations. There were no deaths, intubations, loss of vital signs or other adverse events.  The average length of stay was 12 h 46 minutes and 11.5% of patients were admitted to an inpatient unit. At 30-day follow-up, there were no significant missed injuries. Or re-admissions. | **Madsen et al.** |
| To determine the efficacy and safety the  EDSSU/AMU. The unit was opened every year from November to march. It was mainly used A recruitment for rapid clinical stabilisation of patients with seasonal acute exacer bation of chronic cardiac or respiratory pathologies(or other moderate serious entities.  Most patients admitted were patients with COPD, acute heart failure (77.6%) other diagnosis included: pneumoniae (4,3%), Diabetes mellitus (2,6%), pharmacological overdose (2,6%), acute nephritis (1,0%), acute anaemia 1,1%), acute enteritis (1,7%) and others.  Specifically for COPD patients they found a shorter LOS but a higher readmission rate compared to patients admitted to a normal ward.  a first logical explanation for the shorter mean length of stay is that patients chosen for admission to the EDSSU presented to the emergency department with less severe clinical parameters. However, given that a high percentage of patients admitted to the EDSSU presented with serious illness, the authors  not dismiss other factors that might have also significantly contributed to patient manage ment—for example, strictly focusing on the rapid stabilisation of patients or providing care continuously 24 h a day. Furthermore, the selection of this group of ‘‘less serious’’ patients for admission to the EDSSU did not seem to have had a negative effect on the total average length of stay of the other patients admitted to standard hospital units. | **Juan et al.** |
| Aim of the study:  To investigate the impact of commonly reported ED, EDSSU and hospital census data on the performance of ED reported KPIs (key performance indicator) of NEAT(national emergency access target = LOS in ED < 4 hours) and DNW (did not wait for treatment) rates.  The study found a negative correlation between NEAT performance and DNW (Did Not Wait) rates, suggesting that better NEAT performance could reduce waiting times and DNW cases. During times of high occupancy, ED beds become limited, leading to longer lengths of stay (LOS) for patients awaiting hospital admission. The study recommends treating patients with complex needs outside of the ED for better outcomes. | **Shetty et al.** |
| Determine adequacy of discharge planning from emergency short-stay unit (ESSU), and patient knowledge of and satisfaction with the ESSU.  Conclusions: Most discharged, are provided with adequate discharge instruction. Sizeable proportion require subsequent medical care for the same problem after discharge. Patient satisfaction with all aspects of care in an ESSU is high | **Arendts et al.** |
| Asses beneficial and harmful effects of short-stay unit hospitalisation compared with usual care in people with internal medicine diseases and conditions  Conclusion: quantity and certainty of the evidence was very low. Consequently, it is uncertain whether there are any beneficial or harmful effects of short-stay unit hospitalisation for adults with internal medicine diseases and conditions | **Strøm et al.** |
| Pilot: acceptability of short-stay model for treatment of VOC in SCD outside of the ED Secondary objectives:  patient satisfaction, barriers to its use, comparison of clinical outcomes  Conclusion: high patient satisfaction and acceptability of a short-stay model for treatment of uncomplicated VOC in adult SCD patients | **Binding et al.** |
| Evaluate effect of direct admission to AGU on LOS and  morbidity of elderly patients  Conclusion: Direct admission is associated with shorter hospital LOS and fewer postacute care transfers.  No signifcant association with readmission to the ED <30 d, or with ICU transfers was found. . | **Naouri et al.** |
| to compare admission to the short stay operated  by EM (short stay EM) physician with admission to both the ESSW operated  by other physicians (short stay other) and the general ward (GW)  Conclusions: Short stay-EM significantly reduced ED length of stay compared to other groups  Mortality was lower in both Short stay-EM and short stay other | **Moon et al.** |
| to compare the incidence of functional deterioration of elderly patients  hospitalized in acute care geriatric units compared to that in a conventional care unit  Conclusions: the elderly patients admitted to a geriatric care unit showed less functional deterioration on discharge  compared with those kept in another care unit of a conventional type. | **Zelada et al.** |
| To compare patient and system outcomes before and after introduction of an Acute surgery unit retrospectively  Conclusions: Institution of an ASU was associated with decreased time from referral to  theatre and reduced length of stay. | **Kinnear et al.** |
| to evaluate the management of SBO  before and after introduction of Acute surgical unit (ASU).  The ASU introduction resulted in a statistically  significant reduction in mortality, increased proportion of surgically  managed patients and decreased proportion of patients operated  5 or more days after admission or referral. | **Musiienko et al.** |
| 153-patient randomized trial comparing  observation unit care that included early  cardioversion with routine hospitalization in  patients with uncomplicated atrial fibrillation of less  than 48 hours’ duration.  Patients treated in the observation unit had  substantially shorter hospitalizations and were 12% more likely to be discharged in sinus rhythm. | **Decker et al.** |
| The study aimed to compare the impact of a short-stay geriatric unit versus standard geriatric care on 1-month readmission rates and LOS.  Conclusion: The UTAG reduced length of stay without increasing 1-month readmissions, particularly benefiting frail older patients. | **Moyet et al.** |
| To compare the costs and cost-effectiveness of specialist geriatric care with standard care in frail older adults post-discharge.  Conclusion: The specialist geriatric medical intervention for frail older people discharged from acute medical unit was not cost-effective | **Tanajewski et al.** |
| The study aimed to explore the impact of adding a physician at triage and comparing two additional models of care (PAT and PATplusMAU) with standard care | **Elder et al.** |
| Assess whether consultant supervision is beneficial or not.  two groups: weekdays when the consultant was (Monday, Tuesday, Thursday and Friday) and was not (Wednesdays and other weekdays when the consultant was on annual leave) present on the unit. | **McNeill et al.** |
| To determine the impact of a protocol driven EDOU on LOS, cost and resource utilisation | **Perry M et al.** |
| Intervention:  Emergency Short Stay Ward (ESSW)  Comparison:  Admission general ward | **Ok M et al.** |
| Intervention:  Emergency Department Short Stay Unit (ESSU)  Comparison:  inpatient medical ward or ICU care | **Downes MA et al.** |
| Intervention:  Access to an Acute Geriatric Unit model of care delivered by interdisciplinary teams during acute illness to prevent functional decline and related complications  Comparison:  Usual care | **O’Shaugnessy et al** |
| Short-Stay Unit (SSU) hospitalisation versus internal medicine department (IMD) hospitalisation.  SSU is a multipurpose unit accommodating patients with no life threatening conditions and perceived to be dischargeable <72 hours.  Chief physician is internal medicine specialist  Interventions group:  Primary assessment at ED. Further diagnostic tests on SSU on fast-track basis. Discharge planning initiated immediately.  Comparison group:  IMD care as usual | **Strom C et al.** |
| Survey amongst 362 noninstitutional general and short-stay hospitals  Retrospective evaluation of performance of EDs with and without observation units (OU).  36% of EDs has an OU in the ED | **Wiler et al.** |
