# Supplementary material for: Optimizing efficiency in the acute care chain: a systematic review on the implementation and impact of interdisciplinary short-term monitoring in acute care units
Source: Intern Emerg Med. 2025 Nov 20;21(1):283–95. doi: 10.1007/s11739-025-04194-w (PMC12948887; doi:10.1007/s11739-025-04194-w)
Supplement: Supplementary file 3 — Supplementary file4 (DOCX 2615 KB) [file 11739_2025_4194_MOESM3_ESM.docx]

Supplemental table 2: Study design and characteristics

| Author | Year | Country + City | Patient population  (medical/trauma/exclusions) | Brief summary | Study design  (pre/post, comparison same other hospital) | Definition and collection of outcomes, especially readmission, revisit and /or mortality | % admitted and discharged after observation in unit | Characteristics unit (stand alone, dedicated or shared staff, number beds, staff-patient ratio, occupancy rate) | Characteristics hospital (volume, facilities, number pts ED) | Quality study based on GRADE |
| --- | --- | --- | --- | --- | --- | --- | --- | --- | --- | --- |
| Lévesque et al.    (8) | 2019 | France | 20.3% <60 years  23.1%>60 <75 years  21.3% >75  <85 years  35.3% >85 years (UHA)  43% <60 years  19.9%>60 <75 years  15.5%>75 <85 years  21.6% >85 years  (UHCD)  52.5% female (UHA)  54.3% female (UHCD) | Pre-hospitalization unit (UHA) that functions as a buffer during peak hours, 20 places  And an AMU (UHCD) 16 places  conclusion: The waiting hospitalisation unit at Rouen University Hospital improved emergency patient flow, but weekend bed shortages and epidemic management remain challenges. | Retrospective observational study | Mortality, Return home | UHA  Return home: 23.3%  Transfer to another institution: 6.4%  Discharge against medical advise: 0.2%  Transfer to MCO (conventional hospitalization sectors): 66.3% | Bed manager positions in the adult ED , aided by bed availability software  Medical supervision is provided 365 days a year, by two senior doctors (an emergency doctor, an internist during the week, only one emergency doctor or internist at the weekend) and 3 interns in emergency medicine. | University hospital | **Low-moderate**  Potential selection bias: groups based on availability and departmental needs |
| Conway et al.  O’Riodan, silke    (9) | 2013 | Ireland (Dublin) | all emergency medical admissions,  50.9% female | the Acute Medical Admissions Unit (AMAU) was established in 2003; now, they are retrospectively reviewing all patients, including those from the Intensive Care Unit (ICU) and High Dependency Unit (HDU), from 2002 to 2012 to assess the differences between 2012 and 2002.  Conclusion: AMAU model resulted in significant long-term reductions in in-hospital mortality, length of stay, and emergency department wait times, | Observational cohort study | In-hospital mortality, LOS,  ED waiting time | n/a | n/a | Secondary care centre | **Moderate to high**  Possible selection bias: there is no mention of how patients were selected |
| Moloney et al.    (10) | 2007 | Ireland (Dublin) | all emergency medical patients  (10% >80 years), 52% female | Acute medical admissions unit (AMAU) versus various non-specialty wards. | Retrospective analysis | LOS,  Readmission 28 days | n/a | n/a | Acute teaching hospital | **Moderate**  Potential selection bias: outcomes could change over time (2004 compared with 2002) |
| Moloney et al.    (11) | 2005 | Ireland (Dublin) | all emergency medical patients  (10% >80 years) | Acute medical admissions unit (AMAU) versus various non-specialty wards.  Conclusion: AMAU reduces LOS, waiting time and costs | Retrospective analysis of data recorded in the hospital in-patient enquiry (HIPE) system. | ED waiting time, LOS, Consultant practice, Costs | n/a | n/a | Acute teaching hospital | **Moderate**  Potential selection bias: outcomes could change over time |
| Li et al.  (12) | 2010 | Australia, Adelaide | All admissions to general medical service , 56% female | Comparing general care before a functional AAU and after a functional AAU  Conclusion:The introduction of an Acute Assessment Unit (AAU) led to significant improvements in performance, including shorter hospital stays and an increase in direct discharges, without an increase in hospital mortality or unplanned readmissions | Retrospective comparison | Mean LOS, ED waiting times,  Hospital mortality | AAU to home or setting outside within 24H increased from 13.2% to 17.7% | Twice a day within the AAU, the consultant physician on duty reviews all new admissions. | Tertiary teaching hospital | **Moderate**  Retrospective design  Possible selection bias:  Possible change in referral patterns |
| van der Linden et al.  (13) | 2013 | Netherlands | regular admission patients | Data from a 4-month control period in 2008 and a 4-month intervention period in 2009. During the intervention period, an FAAU with 15 inpatient beds was implemented for off hours.  Conclusion: The FAAU reduced ED crowding by cutting patient boarding and preventing longer ED stays. | before-and-after interventional study | Transfer to other hospitals, LOS-ED | Transfer to other hospital because lack of space  Control period: 10.42%  Intervention period: 6.35% | Admissions coordinator appointed to pay regular visits to the admission floors to scout for empty beds. | Inner-city level 1 trauma center with 50000 visits per year | **Low to moderate**  Single centre and before-after design  Potential selection bias due to seasonal differences and exclusion weekend admissions |
| Budde et al.    (14) | 2018 | New York | All COPD-related ED visits were included for patients aged 40 years or older | Compared the total number of hospital admissions before and after the availability of an Observation Unit (OU), as well as the number of patients discharged directly from the Emergency Department (ED).  Conclusion: The introduction of an Observation Unit (OU) for managing AECOPD led to a significant and immediate reduction in COPD hospital admissions while maintaining stable discharge rates from the Emergency Department | Retrospective cohort study | LOS-ED, Hospital admission rate | 79.6 % of patients were admitted; after the unit was available: 66.8 % were admitted, meaning 20.4 % vs 33.2 % were discharged without admission. | n/a | Tertiary care academic medical center | **Low to moderate**  Retrospective design  Possible selection bias: allocation based on bed availability |
| Meschi et al.  (15) | 2016 | Italy, parma | IM ward generally admits from the ED patients with medical problems who are 65 or older, with multimorbidity, polypharmacy and disability status, frequent hospital admissions who have medical devices or who seek medical care for complex social reasons.  51-58% female | Comparing the IM and come’n’go ward of Internal Medicine and Critical Subacute Care Unit with other units  Conclusion: The geriatric care model reduces in-hospital mortality and length of stay, but does not significantly lower 30-day readmission rates. | Case study | 30 day readmission,  LOS-in-hospital | Transferal to CSC/INMSC wards    Internal Medicine and Critical Subacute Care Unit—Come’n’go and IM wards  2012: 31%  2013: 28%  2014: 25%  Other acute-care internal medicine units  14%  10%  8% | come’n’go ward:  2 hospitalists and some residents normally on duty in the other wards of the unit; nurse-patient ratio 1:6  High-intensity fast-turnover internal medicine ward (IM):  6 hospitalists and some residents; nurse:patient ratio 1:6  Intermediate-intensity critical subacute care ward (CSC):  4 hospitalists and some residents; nurse:patient ratio 1:13  Integrated nurse-managed subacute care ward (INMSC):  senior head nurse and 2 skilled case-manager nurses with medical staff from other wards; nurse:patient ratio 1:9 | University hospital | **Low**  Potential selection bias:  No randomization, no control group, no blinding  Single centre  Observational design |
| Strøm et al.    (16) | 2017 | Denmark | >75 years, 56% female SSU  51% female IMD | Short-Stay Unit (SSU) hospitalisation versus internal medicine department (IMD) hospitalisation.  conclusion: Patients aged 75 and older had fewer adverse events in a short stay unit than in an Internal Medicine Department.  Evaluating adverse events. | Observational study | 90 day mortality, Adverse events, Readmission  (LOS reported but not considered an outcome because short LOS is believed to be a goal) | 90.9 % of the patients admitted to the short-stay unit were discharged successfully (i.e. within 24 h) under the “appropriate admission” criteria | n/a | Secondary referral hospital | **Moderate**  Potential selection bias: two groups based on triage status |
| Flood et al.  (19) | 2013 | USA | Hospitalists' patients aged 70 years or older spending the entirety of their hospitalisation in either the ACE or UC unit in fiscal year 2010. | Examine variable direct costs from an interdisciplinary ACE compared with a multidisciplinary usual care (UC) unit.  Conclusion: The ACE unit team model reduces costs and 30-day readmissions. | Retrospective cohort study. | Costs, Readmission 30 days, LOS | n/a | n/a | Tertiary care academic medical center  1146 bed space | **Moderate - low**  Potential selection bias: patients assigned to units based on bed availability |
| Plamann et al.  (20) | 2018 | USA, minnesota | Adult patients with observation status (typically less acute than regular inpatients) | Implementation of an observation unit to improve patient flow, reduce costs, and enhance patient satisfaction. Pre-implementation of the observation unit vs. Post-implementation  Conclusion: The establishment of an adult observation unit improved care for patients and families through an evidence-based, interdisciplinary approach that enhanced outcomes like LOS and fostered care innovation. | Case study of a hospital unit implementation (observation unit) | LOS, Direct and total costs, Number of patients diverted,  Readmission 72 hours | “The hospital is also seeing fewer patients diverted than prior to the observation unit opening” | Nursing staff: 16.97 full-time equivalent (FTE) registered nurses (RNs) and 10.18 FTE patient care assistants were hired to staff the 14-bed unit. The RNs worked in shifts of 8 or 12 hours, with a nurse-to-patient ratio of 1 RN to 4 patients. A charge nurse also managed two patients while serving as a resource for the unit.  Physicians: A hospitalist physician was present from 8:00 am to 5:30 pm, and after hours the unit was staffed by an advanced practice registered nurse (APRN).  Coordinator: The unit had a nurse coordinator, who split time between clinical duties and operational management, including staffing, appraisals, and performance monitoring. | Large teaching hospital | **Very low**  Risk of bias high no control group or randomization and no formal study design  No pre vs. post data available  No statistical analysis |
| Norman & Sinha  K  (22) | 2022 | Canada, toronto | >65 years  60.7% female | ACE patients were assigned to either the ACE unit, designed specifically for their care, or were distributed to other units. | retrospective cohort study | *Primary outcomes*  Discharge disposition, In-hospital mortality  *Secondary outcomes*  LOS | “patients admitted to the ACE unit were more likely to return home than those who were bed-spaced “  (OR 1.23 [1.02, 1.50], p = 0.033) | five general internal medicine teams | Urban academic health sciences center | **Moderate**  Potential selection bias: decision made where to admit patients made by non-clinical staff |
| Strøm et al.  (23) | 2018 | Denmark (cochrane review worldwide literature) | >18 years, internal medicine conditions (incl pulmonary/chest pain, AF) | Asses beneficial and harmful effects of short-stay unit hospitalisation compared with usual care in people with internal medicine diseases and conditions  Conclusion: quantity and certainty of the evidence was very low. Consequently, it is uncertain whether there are any beneficial or harmful effects of short-stay unit hospitalisation for adults with internal medicine diseases and conditions | Cochrane review: randomised trials and cluster-randomised trials (14 included) | Primary outcomes:   – Mortality  Secondary outcome:   – Hospital readmission | n/a | n/a | n/a | **Very low**  high risk of bias, heterogeneity between trials, and imprecision of effect estimates |
| Nahab et al.  (24) | 2012 | USA, atlanta | All patients with TIA presenting to the ED over a 18-consecutive month period. 61% female | The comparison is between the pre-ADP period, when all patients were admitted to neurology, and the post-ADP period, where patients were managed via the ADP or admitted based on exclusion criteria or physician discretion.  The patients evaluated during the first 11 months of the study (pre-ADP period) were compared with those evaluated in the subsequent 7-month period after the ADP was instituted (post-ADP period). | “before‒after” cohort study | LOS, Costs, Stroke prevention, readmission | n/a | n/a | University Hospital | **Low**  Before after  Possible selection bias: retrospective patient identification from databases  No adjustment for confounders |
| Perry M et al.  (25) | 2021 | United States of America | All adult patients with an urgent/emergent admit to observation order. Patients who were felt to require care beyond 6 h in the ED but have had a greater than 70% likelihood of discharge within 18–24 were con-  sidered for observation services.  57% female  Obstetric patients excluded | To determine the impact of a protocol driven ED observation unit on LOS, cost and resource utilisation | Retrospective, observational, cross-sectional study in 4 hospitals | *Primary outcomes*  LOS in hours, Total direct costs (USD), Disposition after observation (admission or discharge)  Data were retrieved from hospital cost accounting database | Admission rate  EDOU 12.3%  HMSOU 15.6%  NOU 26.4% | 1. ED observation unit (EDOU) close to ED and managed by EP. No use of inpatient beds 2. Hospital Medicine Observation Unit managed by internal medicine physicians, located remotely from the ED 3. Inpatient beds (Non-observation Unit), care provided by admitting service | Four-hospital academic health system: urban teaching and non-teaching hospital, suburban non-teaching hospital and tertiary care specialty-referral center.  No volumes reported | **Moderate-good**  Potential selection bias, however, several types of hospitals included. Generalisability out of USA? |
| Barnes et al.  (26) | 2012 | USA | >70 years  67.77% female | randomized to the Acute Care for Elders intervention group versus randomized to the usual-care control group. | Randomised Controlled Trial | *Primary outcomes*  LOS, Costs  *Secondary outcomes*  Change in ADL, In hospital mortality, Discharge destination, three -month readmission | ACE  Discharged to home: 5%  Usual care  Discharged to home:  3% | Interdisciplinary team rounds were conducted daily by the medical director (a geriatrician) and a geriatric clinical nurse specialist with bedside nurses, physicians, social workers, and physical therapists. | Private, nonprofit teaching hospital | **High**  Possible bias:  ACE received specialized care while control group did not |
| Fox et al.  (27) | 2012 | n/a | Acutely ill or injured adults, 62% female | ACE versus general care  Conclusion: Acute geriatric care with ACE components significantly reduces falls, delirium, functional decline, hospital stay, and costs, while increasing home discharges, particularly for octogenarians. | Systematic review and meta-analysis | *Primary outcomes*  Functional decline,  Iatrogenic complications  *Secondary outcomes*  LOS, Discharge destination,  Mortality during  Hospitalisation,  Hospital costs,  Readmission | Discharge destination  Home 1.05 (1.01–1.10) 2.69 (.01) 0% (.54)  Nursing home  0.96 (0.80–1.15)  0.48 (.63) 50% (.06) | n/a | n/a | **High**  Heterogeneity was thoroughly assessed, and sensitivity analyses were performed to explore the robustness of the findings |
| Tanajewski et al.  (28) | 2015 | UK | Patients aged 70+ with a score of 2+ on the Identification of Seniors At Risk tool, discharged within 72 hours, were eligible. | To compare the costs and cost-effectiveness of specialist geriatric care with standard care in frail older adults post-discharge.  Conclusion: The specialist geriatric medical intervention for frail older people discharged from acute medical unit was not cost-effective | two-centre randomised controlled trial | **Primary outcome:** Cost of medical and nursing care  **Secondary outcomes:** Associations with education, outings, comorbidities, hospital attendance, and household composition | n/a | n/a | Center 1: 675,000 catchment population  Center 2: 1,1 million catchment population | **Low**  Economic evaluation with uncertainties in cost estimates, generalisability, and resource use assumptions, potential bias in cost measurement and extrapolation. |
| O’Shaugnessy et al  (29) | 2022 | USA (7), Sweden (3), Australia (1) | Older adults (≥65 years) admitted to an AGU with acute medical complaints, not requiring speciality unit | Intervention:  Access to an Acute Geriatric Unit model of care delivered by interdisciplinary teams during acute illness to prevent functional decline and related complications  Comparison:  Usual care | Systematic review  (update of previous meta-analysis) | 1-month hospital readmission, 3-month hospital readmission  Mortality during index admission and 90-days mortality  Recorded at discharge from hospital and at follow-up periods in trials. | N/a | Various models of Acute Geriatric Units:  Bed capacity 10-34 beds  Core interdisciplinary team comprised at least one geriatrician and/or primary physician and nurses with geriatric training.  Physiotherapist included in all units. Social worker in 9/11 and dietitian 4/11, pharmacist 2/11. | Various studies and hospitals | **Low-Moderate**  Selection bias: USA studies  Variation in outcomes leading to limited pooling opportunities |
| Juan et al.  (32) | 2006 | Spain, Barcelona | Admission criteria: Home discharge prediction of >12 h but <72 h; confirmed clear medical diagnosis in the ED before entering the EDSSU  Exclusion criteria: no surgical conditions; no social problems; and no isolation requirements  56% female | To determine the efficacy and safety the EDSSU/AMU, opened every year from November to March.  Most patients admitted were patients with COPD, acute heart failure (77.6%)  Specifically for COPD patients they found a shorter LOS but a higher readmission rate compared to patients admitted to a normal ward. | Retrospective chart review | *Outcomes*  Main activity and quality outcomes of the EDSSU during first 6 years,  Satisfaction of patients treated in the EDSSU  Diagnostic-Related Groups and Clinical Characteristics Comparison  Mortality and readmission  Transfers to other units | Only mentioned for the short stay unit  Transfer to hospital units  First year: 8.5%  Second year: 8.5%  3: 9%  4: 6%  5: 7%  6: 6% | 24 beds with an attending doctor assigned to the EDSSU 24 h/day, with three shifts on weekdays (first shift, 8:00–16:00 h; second shift, 12:00 20:00 h; third shift 20:00–8:00 h) and a 24-h shift on Saturdays and Sundays. The EDSSU also has two consulting specialists from the respiratory and cardiology services assigned specifically to the unit.  The staff of the AMU was additional to normal hospital staff, with a special budget for the unit. | Tertiary-care teaching institution | **Low-Moderate**  Selection bias: COPD patients only, EDSSU only open Nov-March results may not reflect outcomes outside these months |
| McNeill et al.  (33) | 2009 | UK | Admissions to the AMU at Ipswich Hospital from January 1, 2005, to August 31, 2005.  Weekends and bank holidays data were excluded due to limited staffing.  56-59% female | Assess whether consultant supervision is beneficial or not.  two groups: weekdays when the consultant was (Monday, Tuesday, Thursday and Friday) and was not (Wednesdays and other weekdays when the consultant was on annual leave) present on the unit. | retrospective cohort study | **Primary outcome:** Length of stay (mean days) & same-day discharge rate  **Secondary outcomes:** Readmission (30, 60 days), in-hospital mortality | 32.2% discharged on the same day when consultant present vs 23.0% when absent | a single consultant presence on four days out of five during the working week. The consultant would be present on the unit from 09.00 until 17.00. | general hospital | **Low**  Single centre observational design with potential confounding and selection bias; no randomisation; limited adjustment for case mix; outcomes likely influenced by factors beyond consultant presence. |
| Kinnear et al.  (34) | 2017 | Australia, Adelaide | Adult patients admitted under a general surgical team and underwent a non-elective general surgical procedure.  53% female | To compare patient and system outcomes before and after introduction of an Acute surgery unit  Conclusions: Institution of an ASU was associated with decreased time from referral to  theatre and reduced length of stay. | A retrospective historical control study (before and after) | 1) Primary outcomes Median time from emergency department (ED) referral to thea-tre start; Proportion of cases managed in hours (08.00–18.00); Median total length of stay  2) Secondary outcomes: Admission to ICU; Representation to ED within 30 days of procedure; In-hospital mortality; 1-year all-cause mortality | n/a | Acute Surgical Unit, consultant led.  On-site registrar. Rotating surgeon consultant on-call with no elective commitments.  Weekday support by surgical sub-specialty junior and senior registrars.  Emergency theatre for use of ASU. | Tertiary referral hospital | **Low**  Impact availability of emergency theatre versus ASU unclear. Bias on all-cause mortality given missing data (in-hospital mortality known); 20% patients excluded due to missing data |
| Conway et al.  bruyne, cournane  (35) | 2018 | Ireland, Dublin | 51% female | Comparison before and 15 years after the opening of an AMAU. Meanly comparing high risk patients vs low risk | Retrospective observational cohort study | 30day-in hospital mortality, LOS,  Readmission | n/a | n/a | Secondary care centre | **Moderate**  Retrospective and single centre  Potential selection bias: no randomization |
| Musiienko et al.  (36) | 2016 | Australia, Melbourne | adult patients with a diagnosis potentially related to Small Bowel obstruction (SBO).  50% female | to evaluate the management of SBO  before and after introduction of Acute surgical unit (ASU).  The ASU introduction resulted in a statistically  significant reduction in mortality, increased proportion of surgically  managed patients and decreased proportion of patients operated  5 or more days after admission or referral.  LOS did not change | A retrospective review, before and after study | Overall mortality, in-hospital LOS, 30-day readmission, ICU admission  Data collected from the hospital administrative database and general surgical specialties’database. | n/a | Acute surgical unit, localised with dedicated space in acute assessment unit. A dedicated consultant surgeon was on-site from 07.30 to  18.00 h and led a team treating emergency general surgical patients only. | Major tertiary care centre. | **Low**  Selection bias: only short bowel obstruction patients, limited volume |
| Elder et al.  (37) | 2016 | Australia | All presentations made to the ED over a six-week period in 2013 compared to the same period in 2012  50% female | Exploration of the impact of adding a physician at triage and comparing two additional models of care: physician at triage (PAT) and PATplus medical assessment unit (MAU) with standard care | A comparative , before and after, retrospective study | *Outcomes*  Time to see an ED health care professional; ED-LOS;  Number of Admissions;  The number of patients who presented to ED and left before medical assessment; Re-presentations <48 hours to the ED, The percentage of patients assessed and discharged or admitted to ward environments within 4 hours of presenting to the ED  Data were extracted from the ED information system | Standard care 34%  PAT 38%  PATplusMAU 40%  No differentiation between admission at ward or MAU | The MAU was staffed by a pool of 51 full-time equivalent nurses and nine doctors (five senior doctors and four interns)  PAT model of care, the staffing consisted of:  Medical Officer: Reallocated from existing ED staff to the triage area.  Triage Nurses: One or two nurses working alongside the medical officer. | 570-bed regional, public teaching hospital  494,501 catchment population  74,277 ED presentations per year | **Moderate**  Retrospective study, datahandling suboptimal . Studydesign affecting generalisibility |
| Candelli M et al.  (38) | 2023 | Italy, rome | Patients with gastrointestinal bleeding.  Excluded patients referred by spokes centres | The study compares outcomes and resource use between patients in regular wards and those in an SSU.  Conclusion: Treating NVUGIB in the SSU led to shorter hospital stays, quicker endoscopy times, and fewer transfusions. | Retrospective, single-centre observational study | Hospital mortality  Readmission 30 days, LOS | n/a | Medical staff of our SSU consists of all the doctors of ED, who take turns to take care of the patients admitted there, and a chief physician with expertise in gastroenterology | Private university hospital | **Moderate**  Retrospective design  Potential selection bias: admissions SSU based on specific criteria |
| Russell PT et al.    (39) | 2014 | Australia | All general medical patients admitted to the SSU | Assess the impact of an SSU on the efficiency of the department by comparing SSU versus general ward and LSU (long stay unit)  Conclusion: An SSU for general medical patients is a safe alternative to a traditional ward, but it does not significantly enhance overall efficiency or expedite discharge compared to normal care. | Retrospective study | Relative stay index,  Inhospital mortality,  Readmission 7 and 28 days | n/a | Staffed on a week-by-week basis by one of the consultant general physicians accompanied by an intern, resident medical officer and medical registrar | Tertiary hospital  5000 ED patients per year | **Moderate**  Retrospective design  Study includes misallocated patients |
| Yong et al.  (40) | 2011 | Australia | All general medical patients admitted to the SSU.  56% female | To evaluate their ability to predict a patient’s likely length of stay (LOS) And to describe differences between patients who were allocated inappropriately to either the long-stay unit (LSU) (i.e. LOS < 72 h) or to the SSU (LOS>72 h). Finally, evaluation of the quality of care afforded to short-stay patients  the establishment of a medical SSU at FMC allowed accurate identification of a stream of patients, who could be treated safely without compromising patients’ outcomes. The accuracy of prediction of long and short-stay patients improved over time. | retrospective observational study | LOS, (ED LOS not included)  Readmission, Mortality, Transfer ICU | Transfer to ICU  2005: 3%  2006: 2%  2007: 1%  2008: 1% | 12 bed medical SSU (short stay unit) was staffed by dedicated nursing staff and a consultant general physician and three trainee medical officers (TMOs) from Monday to Friday.  On the weekends, the ‘‘on call physician’’ and one TMO attended the new admissions to the unit.  Senior consultant bedside <12 hours. | 500-bed tertiary referral, university teaching hospital | **Low-moderate**  Retrospective design  Single centre  Potential selection bias:  Allocation based on consultant judgment |
| Cheng et al.  (41) | 2016 | Canada | The post-OU cohort included all adults presenting 6 months after OU implementation. The pre-OU cohort included all adults presenting in the same 6-month period 1 year before OU implementation.  Pre-OU= 52.5% female  Post-OU= 53.4% female  ED B  Pre-OU= 50.6% female  Post-OU= 51.6% female | Evaluation of two hospitals before OU implementation and after OU implementation  Conclusion: OU implementation decreased hospital admissions at one site but did not reduce ED LOS for all patients. | two-center, pre–post study | *Primary outcomes*  ED LOS, Hospital admission rate  *Secondary outcomes*  % ED assigned to OU, ED LOS of OU patients, % inhospital admissions from OU, Readmission within 72H | Hospital admission rate  Hospital 1  Pre OU: 17.8%  Post OU: 17%  2  Pre OU: 18.9%  Post OU: 18.3% | OU nurse (ED nurses trained in OU function and protocols) and the attending EP using standardized order sets and protocols  designated physician responsible for the OU patients at all times who also had the responsibility of managing regular ED patients during the shift. Emergency physician staffing resources were increased at ED B (by 5 h/d) to care for OU patients, but not at ED A during the study period | Hospital 1: local referral center with 62000 ED visits a year  Hospital 2: academic regional trauma center with 67000 ED visits a year | **Moderate**  Potential selection bias: not randomized  No multivariable corrections noted |
| Mong et al.  (42) | 2017 | Singapore | Patients 12 years or older who have features of poisoning that require treatment or extended observation and be deemed likely to improve within 24 hours. Exclusion criteria: patients (1) with unstable vital signs after initial treatment in the ED, (2) with evidence of end-organ injury and (3) have other features requiring management in the intensive care unit or high dependency unit.  59% female | Goal: to describe the management of patients with toxic exposure in a short stay obersvation unit.  Conclusion: Most patients (93%) admitted to the EDOU (emergency department observation unit) were successfully managed and medically cleared within 23 hours, including those with a severe PSS. (poison severity score due to a GCS 8 or less). Previous studies showed a local LOS on a normal ward of 3 days. Admission on a AMU/EDOU could decrease the LOS for patients with toxic exposure | Retrospective chart review | LOS, Discharge,  Readmission | 69.9% home  22% psychiatric unit  5.6% inpatient admission | n/a | EDOU in eastern Signapore  150 000 ED attendances a year | **Low-moderate**  Retrospective design,limited generalizability |
| Binding et al.  (43) | 2019 | Canada | Adult (>18 years) sickle cell patients with uncomplicated veno-occlusive crisis  76% female | Pilot: acceptability of short-stay model for treatment of VOC in SCD outside of the ED Secondary objectives: patient satisfaction, barriers to its use, comparison of clinical outcomes  Conclusion: high patient satisfaction and acceptability of a short-stay model for treatment of uncomplicated VOC in adult SCD patients | before-and-after pilot study (observational) | *Primary outcome*  Time from patient arrival to administration of first opiate dose  *Secondary outcome*  Length of stay,  Total opiate dose per hour,  Pain scores,  Disposition status | 15.8% transfer to GW | nursing and medical staff trained in the management of uncomplicated VOC. | Tertiary care | **Very Low**  Selection bias only inclusion of sickle cell disease with moderate vaso-occlusive crises  Only 12 unique patients |
| Moon et al.  (44) | 2023 | South-Korea, Seoul | adult patients who had visited the adult ED and admitted  41-47% female | to compare admission to the short stay operated  by EM (short stay EM) physician with admission to both the ESSW operated  by other physicians (short stay other) and the general ward (GW)  Conclusions: Short stay-EM significantly reduced ED length of stay compared to other groups  Mortality was lower in both Short stay-EM and short stay other | retrospective observational study | *Primary outcomes*  ED length of stay, 28-day hospital mortality  *Secondary outcomes*  Hospital length of stay, Type of discharge, ICU admission | ICU admission  ESSW-EM 5.8%  ESSW-Other 4%  GW 7%  Discharged  ESSW-EM 73.5%  ESSW-Other 85.2%  GW 84.5%  Transferred to other hospital  ESSW-EM 21.4%  ESSW-Other 7%  GW 1.6%  Staying at the hospital  ESSW-EM 3.3%  ESSW-Other 5.6%  GW 9.8%  Deceased  ESSW-EM 1.9%  ESSW-Other 2.2%  GW 4.1% | Emergency physicians or physicians from other departments | Tertiary academic hospital | **Moderate**  Retrospective design  Potential selection bias:  Admission to ESSW not random |
| Decker et al.  (45) | 2008 | USA | Adult patients presenting to the ED with atrial fibrillation of less than 48 hours’ duration without  hemodynamic instability or other comorbid conditions  39% female | 153-patient randomized trial comparing  observation unit care that included early  cardioversion with routine hospitalization.  Patients treated in the observation unit had  substantially shorter hospitalizations and were 12% more likely to be discharged in sinus rhythm. | prospective randomized study | *Primary outcomes*  Conversion to sinus rhytm/rate control  *Secondary outcomes*  Recurrence of AF or adverse events, Hospital revisits <6 months  Data were collected by telephonic follow-up in addition to record review | n/a | ED  management was at the discretion of the emergency medicine  attending physician on duty.  No characteristics of the ED observation unit were reported | Tertiary referral center  252,392 ED-visits | **Low**  Selection bias: only atrial fibrillation patients, single center, few inlcusions |
| Ok M et al.  (46) | 2020 | Korea | All patients admitted via the ED.  Exclusion of ICU patient, hematological patients and patients with DNR  53-58% female | Intervention:  Emergency Short Stay Ward (ESSW)  Comparison:  Admission general ward | Retrospective observational study, used prospectively collected data.  Matched analysis in one hospital | *Primary outcome*  ED boarding time  *Secondary outcome*  30-day in hospital mortality  Data collected from in-house data retrieval system | 100% admitted: targeting patients only after decision to admit to a general ward | ESSW located above ED, administratively separated from ED.  Maximum admission duration 48h. Located above the ED.  25 beds, 3-room facility. 3 nurses during each shift. Patients are cared for by specialists, admission criteria and managed conform a general ward. | Level 1 ED urban tertiary teaching hospital  2200 inpatient beds  100,000 ED visits per year, 25% admitted | **Low**  Risk on bias given study design, effectiveness during high bed occupancy demonstrated. |
| Downes MA et al.  (47) | 2017 | Australia | Intoxicated adults,  63% female  No critical care, immobilised patients or children. | Intervention:  Emergency Department Short Stay Unit (ESSU) comparable to AMU  Comparison:  inpatient medical ward or ICU care | Retrospective before and after study of all poisoned patients admitted to a tertiary toxicology service via the ED | *Primary outcome*  LOS- ED  *Secondary outcome*  28 days re-presentations at the ED  Data extracted from the toxicology database supplemented with ED data | 67% discharged home  28-30% discharged to psychiatric facility  4% other disposition | ED-SSU with 12 beds with 3 dedicated nursing staff members during daytime and 2 during evening and overnight. Medical officer allocated for each shift.  Admission 24 hours a day, no discharge at night.  ED nursing staff authorised to allocate a bed in the ED-SSU | Tertiary referral hospital with toxicology service.  Urban district ED, annual census 36,000  620 000 catchment population | **Moderate**  Selection bias: specific patient population. Potential same effect by enhancing number of inpatient beds |
| Wiler et al.  (48) | 2011 | United States of America | All ED patients, exclusion: ICU patients, cardiac catheterization laboratory or Operating rooms.  49-56% female | Survey amongst 362 noninstitutional general and short-stay hospitals  Retrospective evaluation of performance of EDs with and without observation units (OU).  (36% of EDs has an OU in the ED) | Retrospective, cross-sectional multicenter cohort study (4-week survey) | *Primary outcome*  Disposition: home / ED observation / short-stay hospital admission  Short stay hospital admission defined as admission to inpatient floor, mental health/detoxification or telemetry bed and hospital LOS <48 hours. | 2.1% admission to ED observation (69% ED managed, 23% inpatient managed)  13% admission hospital bed  15% of all ED patients were admitted, with 27% either short stay of ED observation | Various organisational characteristics:  ED-managed OU,  ED with inpatient managed OU.  ED without OU. | Not reported | **Moderate**  Selection bias in responders versus non-responders, missing data on some outcomes |
| Ribbink et al.  (49) | 2021 | Europe  Spain, barcelona  Netherlands, amsterdam | Admission criteria to both units were:  1.  acute medical problems in older patients that require hospitalization  2.  hemodynamic stability;  3.  no need for complex diagnostic testin;  4.  expecting to return to previous living situation in 10–14 days; and  5.  only for AGCH: geriatric conditions  SCU 60% female  AGCH 51.7% female | SCU vs AGCH  Conclusion: acute geriatric units in two settings are similar alternatives to general hospitals. | Exploratory cohort study.  Gathered date from existing prospective cohort studies | Discharge,  Mortality,  Readmission | Discharge to original living situation:  SCU: 76.1%  AGCH 79.9% | *SCU*  Daytime staffing includes one geriatrician per 12-14 beds, with a total of 3 geriatricians, and one registered nurse per 12-14 beds, totaling 3 nurses. Additionally, there is one nurse assistant per 10 beds and one physiotherapist per 40 beds. A social worker is also available, with one assigned per 40 beds.  For evening and night shifts, there is an on-call geriatrician from the same facility attending the SCU. Registered nurses are staffed at a ratio of 1 per 20 beds, with 2 on duty, and there are 2 nurse assistants also at a ratio of 1 per 20 beds.  *AGCH*  The daytime team includes one geriatrician or internist per 23 beds, with a total of 1 geriatrician or internist. Additionally, there is one nurse practitioner per 8-10 beds and 4 registered nurses, with one nurse per 4-6 beds. There is one nurse assistant per 23 beds and two physiotherapists, with one physiotherapist per 10-12 beds. No social worker is listed in the staffing.  In the evening and night shifts, an on-call geriatrician or internist from a separate facility attends the AGCH. Registered nurses are staffed at a ratio of 1 per 10-12 beds, with 2 on duty, and there is 1 nurse assistant per 23 beds. | Spain: 350-bed intermediate care hospital  Netherlands: 123-bed skilled nursing facility | **Moderate-high**  Potential selection bias: difference in data collection between the centres |
| Naouri et al.  (50) | 2022 | France, paris | patients≥75 years, admitted to an AGU (acute geriatric unit) for more than 24 h  67% female | Evaluate effect of direct admission to AGU on LOS and morbidity of elderly patients  Conclusion: Direct admission is associated with shorter hospital LOS and fewer postacute care transfers.  No signifcant association with readmission to the ED <30 d, or with ICU transfers was found. | Retrospective cohort study  (database, 12 AGU’s) | *Primary outcome*  Hospital length of stay (LOS)  *Secondary outcomes*  Postacute care transfer at the end of the index hospitalization,  Emergency department (ED) return visit within 30 days,  Intensive care unit (ICU) admission from the AGU | Postacute care transfer  46.6% direct admission to AGU group  50.2% first admission to ED group | Not described | Data from 39 university hospitals | **Moderate-high**  Possible selection bias, exclusion of life-threatening ED presentations |
| Zelada et al.  (51) | 2009 | Peru | patients aged 65 and older,  admitted for an acute medical pathology  42% female | To compare the incidence of functional deterioration of elderly patients  hospitalized in acute care geriatric units compared to that in a conventional care unit  Conclusions: the elderly patients admitted to a geriatric care unit showed less functional deterioration on discharge  compared with those kept in another care unit of a conventional type. | Prospective controlled observational study | Basic activities of daily living  The length of hospital stay  Cognitive state & affective state | Not reported | The geriatric unit had in attendance one geriatric physician, one medical resident of the specialty, general care nurses, a physical therapist, an occupational therapist and a social worker (this last one only once per week). The internal  medicine care unit was attended by an internist physician, a medical resident of this specialty, general care nurses, and had access to physical and occupational therapy, and a social worker by  means of referral. | Major tertiary care center, academic  78000 ED visits | **Moderate**  83.6% completed treatment  Selection bias: non randomized allocation and broad exclusion criteria |
| Gruenberg et al.  (52) | 2018 | USA | Inclusion: history of chest pain, vital signs in acceptable limits, electrocardiogram without acute ischemic changes, initial cardiac markers within normal range, resolving chest pain, potential to discharge in < 24 h, able to give consent, and i.v. access.  geriatric= 59.6% female  non-geriatric= 50.9% female | Geriatric ( ≥ 65 Years) compared to non-geriatric patients (< 65 Years)  Conclusion:  Geriatric and non-geriatric patients on an EDOU chest pain pathway have similar 30-day re-presentations; geriatric patients experience longer LOS, partly due to more nuclear stress tests. | Exploratory, retrospective, cohort study | *Primary outcome*  Readmission 30 days  *Secondary outcomes*  EDOU LOS,  Stress testing | n/a | n/a | Urban academic ED annually 120 000 patients | **Low**  Retrospective  Single centre  Potential selection bias: no randomisation and stress testing was at the discretion of physicians not protocol driven |
| Bradas C et al.    (53) | 2016 | USA, Ohio | all patients age 18 and older admitted to the SSU | The SSU was compared immediately after opening and 12 months later.  Conclusion: After a year, the SSU had lower 30-day readmission rates for all diagnoses, suggesting better patient selection and improved discharge processes. | Double cohort observational retrospective study | *Primary outcome*  Hospital readmission 30 days | n/a | The 18-bed SSU  is staffed by hospitalists, two senior  residents, registered nurses, customer care partners, a case manager,  and a social worker | 641-bed public academic medical center | **Moderate**  Retrospective design  Single center |
| Strom C et al.  (54) | 2018 | Denmark, Holbaek Hospital. | Older patients, 75 years or older, acutely admitted, green tag triage in ED,  49-54% female | Short-Stay Unit (SSU) hospitalisation versus internal medicine department (IMD) hospitalisation.  Intervention group:  Primary assessment at ED. Further diagnostic tests on SSU on fast-track basis. Discharge planning initiated immediately.  Comparison group:  IMD care as usual | Pragmatic single center, parallel RCT in secondary referral teaching hospital | *Primary outcome:*  90 days mortality after admission, retrieved from Danish Civil Registration Registry  *Secondary outcomes*  Mortality rate from patient inclusion to trial conclusion,  In-hospital mortality,  Adverse events during hospitalisation,  Change in IADL score <90 days form admission,  LOS in hospital,  Unplanned readmission <30 days | Not reported | SSU is a multipurpose unit accommodating patients with no life threatening conditions and perceived to be dischargeable <72 hours.  8 2-bed patients rooms  1 6 chair room  Staff rotating between SSU and ED, with exception of few staff members dedicated to SSU.  Chief physician is internal medicine specialist | Secondary teaching and referral hospital.  270,000 catchment population | **Moderate**  Selection bias: specific patient population (older and green triaged) |
| Arendts et al.  (55) | 2006 | Australia | All patients admitted to an ESSU in one tertiary referral center.  English speaking, able to fill in questionnaire    49-53% female | Determine adequacy of discharge planning from emergency short-stay unit (ESSU), and patient knowledge of and satisfaction with the ESSU.  Conclusions: Most discharged, are provided with adequate discharge instruction. Sizeable proportion require subsequent medical care for the same problem after discharge. Patient satisfaction with all aspects of care in an ESSU is high | Questionnaire study, observational (cross-sectional) | *Primary outcomes*  Discharge planning, Patient knowledge of the ESSU  *Secondary outcomes*  Patient satisfaction with the ESSU,  Readmission,  Comfort,  Communication (information about length of stay, diagnosis, medication, investigations) | Not reported | emergency physician  emergency registrar or consultant  nurses | Tertiary referral hospital | **Low -moderate**  Only 211 responders  3month time for survey |
| Realdi et al.  (56) | 2011 | Italy, padova | Non-trauma and non neurologiocal admissions, *intermediate risk*.  conditions like low-intermediate risk chest pain, heart failure, stable arrhythmias, syncope, high-risk deep vein thrombosis, asthma, pain, and various metabolic or inflammatory disorders.  50% female | Patients with ‘intermediate risk’ medical problems where admitted to a rapid intensive observation unit (RIO). With dedicated and fast diagnostic tools. With the goal of discharging <72u hours.  Outcome divided in RIO patients and RIO-failure (admission to normal ward) | Observational study | *Primary outcomes*  LOS, Discharge rates  *Secondary outcomes*  Patient and staff satisfaction,  Readmission rates | n/a | ‘dedicated staff’, not specified | University teaching hospital | **Low-very low**  High risk of bias due to observational design and lack control  High imprecision due to lack of robust data |
| Grossman et al.  (57) | 2016 |  | Adults (18+) presenting with syncope who were admitted to either inpatient wards or EDOU.  Hospitalized (Full Admission): 52% females  Hospitalized (1-Day Admission): 54% females  EDOU  Patients: 6% females | inpatient wards versus EDOU (Emergency Department Observation Unit)  Conclusion: EDOU patients are less likely to have a diagnosed etiology than admitted patients. | prospective, observational, cohort study | *Primary outcome*  Rate of diagnoses  *Secondary outcomes*  Comorbidities | 49% full admission | A trained research assistant available 16 h per day prospectively screened patients with complaints of syncope or loss of consciousness and reviewed daily patient logs to ensure completion of documentation and to identify missed off-hour patients. | Large urban teaching hospital  Annual ED census of 55000 | **Moderate**  Potential risk of selection bias ]: no randomization |
| Moyet et al.  (58) | 2023 | France | Patients aged 75+ with frailty, comorbidities, requiring short-term care (48-72 hours) and geriatric evaluation post-ED.  67% female | The study aimed to compare the impact of a short-stay geriatric unit versus standard geriatric care on 1-month readmission rates and LOS.  Conclusion: The UTAG reduced length of stay without increasing 1-month readmissions, particularly benefiting frail older patients. | Retrospective descriptive study | In-hospital Length of Stay, 30-days readmission | 60% discharge home after admission, 19% discharge to nursing home, 15% transfer to other unit | 12-bed unit.  The team of the UTAG includes nurses and professional caregivers trained in gerontology as well as physiotherapists and occupational therapists and a social worker. Geriatrician attached to UTAG and ED | 1671-bed university hospital | **Low**  Missing data on readmission possible, ‘quasi experimental before and after study’ |

*** AAU = Acute Admission Unit, ACE = Acute Care unit for Elderly, AGCH = Acute Geriatric Community Hospital, AMAU = Acute Admission Medical Unit, AMU = Acute Medical Unit, CSC = Critical Subacute Care, ED = Emergency department, EDOU = Emergency Department Observation Unit, FAAU = Flexible Acute Admission Unit, HDU = High Dependency Unit, HMSOU = Hospital medical and surgical observation unit, ICU = Intensive Care Unit, INMSC = Integrated Nurse Managed Subacute Ward, LOS = Length of Stay, OU = Observation Unit, SCU= Subacute Care Unit, SSU = Short Stay Unit, UHA = Pre-hospitalization unit.
